# Redox Regulation of K^+^ Channel: Role of Thioredoxin

**DOI:** 10.1089/ars.2023.0416

**Published:** 2024-11-25

**Authors:** Rob H.P. Hilgers, Kumuda C. Das

**Affiliations:** Department of Internal Medicine, Texas Tech University Health Sciences Center, Lubbock, Texas, USA.

**Keywords:** thioredoxin, redox, K^+^ channel, oxidative stress

## Abstract

**Significance:**

Potassium channels regulate the influx and efflux of K^+^ ions in various cell types that generate and propagate action potential associated with excitation, contraction, and relaxation of various cell types. Although redox active cysteines are critically important for channel activity, the redox regulation of K^+^ channels by thioredoxin (Trx) has not been systematically reviewed.

**Recent Advances:**

Redox regulation of K^+^ channel is now increasingly recognized as drug targets in the pathological condition of several cardiovascular disease processes. The role of Trx in regulation of these channels and its implication in pathological conditions have not been adequately reviewed. This review specifically focuses on the redox-regulatory role of Trx on K^+^ channel structure and function in physiological and pathophysiological conditions.

**Critical Issues:**

Ion channels, including K^+^ channel, have been implicated in the functioning of cardiomyocyte excitation–contraction coupling, vascular hyperpolarization, cellular proliferation, and neuronal stimulation in physiological and pathophysiological conditions. Although oxidation–reduction of ion channels is critically important in their function, the role of Trx, redox regulatory protein in regulation of these channels, and its implication in pathological conditions need to be studied to gain further insight into channel function.

**Future Directions:**

Future studies need to map all redox regulatory pathways in channel structure and function using novel mouse models and redox proteomic and signal transduction studies, which modulate various currents and altered excitability of relevant cells implicated in a pathological condition. We are yet at infancy of studies related to redox control of various K^+^ channels and structured and focused studies with novel animal models. *Antioxid. Redox Signal.* 41, 818–844.

## Introduction

**FIG. 1.**
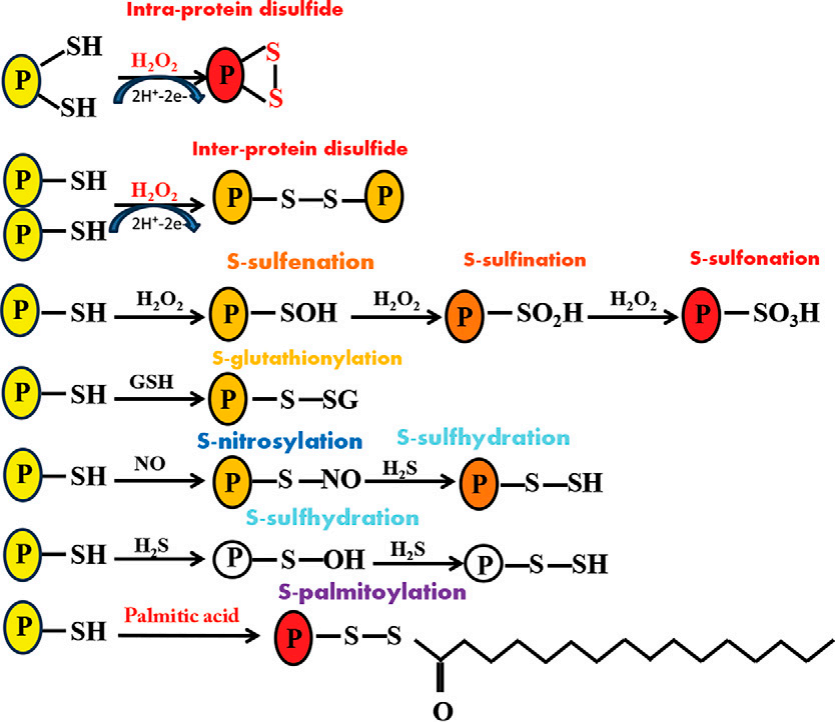
**Examples of protein cysteine sulfhydryl modifications. Hydrogen peroxide (H_2_O_2_) can result in intra- or interprotein (P) cysteine disulfide bridge formation.** In addition, it can cause cysteine residue oxidation reactions depending on the degree of H_2_O_2_ (*e.g.*, S-sulfenation, S-sulfination, and S-sulfonation). Other reducing agents, such as glutathione (GSH), nitric oxide (NO), hydrogen sulfide (H_2_S), or palmitic acid, can result in posttranslational protein S-modifications.

Reactive oxygen species (ROS) are produced and metabolized in all aerobic organisms, and play vital roles in signaling pathways required for normal cellular functions. However, excess ROS production is associated with cellular dysfunction and death (Nordberg and Arner, 2001; Sies et al., 2017; Thannickal and Fanburg, 2000). The steady-state ROS generation is balanced by interaction between ROS and their enzymatic or nonenzymatic antioxidants and scavengers. However, increased levels of ROS production due to altered pathophysiology overwhelm the endogenous defense systems resulting in the onset and propagation of various diseases. For example, increased ROS generation in the vascular cells is implicated in hypertension, ischemia–reperfusion, age-related arterial stiffness, atherosclerosis, cerebrovascular diseases, and other related diseases (Nordberg and Arner, 2001; Wolin, 2009). Cellular oxidants produced due to activation of nicotinamide adenine dinucleotide phosphate (NADPH) oxidases, mitochondria, and exposure to toxic chemicals or gases could cause overt oxidative stress resulting in impairment of normal physiological functions with onset of disease of various organ systems, such as cardiovascular, pulmonary, cerebral, kidney, and many other organs. However, physiological oxidants, such as hydrogen peroxide (H_2_O_2_), produced due to activation of NADPH oxidase or mitochondria could propagate various signaling resulting in protein function or dysfunction. Oxidants react with the thiol group of cysteine (Cys) and with the thioether group of methionine, leading to formation of reversible protein disulfides and methionine sulfoxides, respectively. The human proteome consists of 214,000 Cys residues (Go and Jones, 2013), many of them having the potential to be oxidized by oxidants such as H_2_O_2_ to form disulfides if accessible. These disulfides could be reverted to thiols by various enzymes and low-molecular-weight thiols, such as glutathione or thioredoxin (Trx) (Jerng and Pfaffinger, 2014; Lu and Holmgren, 2014; Nordberg and Arner, 2001; Sengupta et al., 2019; Whayne et al., 2015). About half of all proteins are sensitive to thiol modulation. Examples of Cys thiol modifications are S-glutathionylation, S-nitrosylation, S-palmitoylation, and S-sulfhydration. Recently, polysulfides, including hydrogen per- and poly-sulfides (H2Sn, *n* = 2 and *n* > 2), have been demonstrated as oxidants to S-sulfurate (S-sulfhydrate Cys residues of target proteins, including TRPA1 channels, PTEN, KATP channels, protein kinase G1α, and others (Kimura, 2020). One of the two responsible Cys residues can be S-sulfurated by H2Sn and react with the remaining Cys residues to produce the disulfide bridge. These posttranslational thiol modifications serve as redox-sensing molecular switches (Fig. 1) (Nagahara, 2011). Since oxidative stress could be transient or chronic, some of these modifications are reversible (disulfides, sulfenylation, persulfides, S-thiolations), whereas others are irreversible (sulfinylations, sulfonylations, *etc.*). For example, aging is a chronic oxidation process as we breathe oxygen and metabolize this using an oxidative process in the mitochondria. This chronic slow oxidation process over the life span of an organism promotes dysfunction of various proteins in the vascular system resulting in the onset of several cardiovascular diseases such as hypertension, chronic kidney disease, and various heart diseases. The transient oxidation followed by reduction propagates redox signaling resulting in transcription factor activation or inhibition that is linked to gene expression or suppression. Disruption of these reversible posttranslational modifications is the most central feature of oxidative stress that contributes to mechanisms of aging and age-related disease (Jones, 2008; Jones, 2006). In the cytosol, the redox state is predominantly in a reduced environment such that Cys residues exist mainly in the thiol form rather than in the oxidized disulfide form. Ion channels, specifically opening of potassium channels (K^+^), are regulated by the redox state of their Cys that is involved in various functions in the vasculature, heart, lung, brain, and other organs. Therefore, these channels are impacted by transient oxidations of their Cys and redox signaling. Although these channels are extensively studied, redox regulation of these channels is not adequately reviewed. In this study, we review the redox regulation of various K^+^ channels and their role in cardiovascular and pulmonary systems and diseases.

### Trx system

**FIG. 2.**
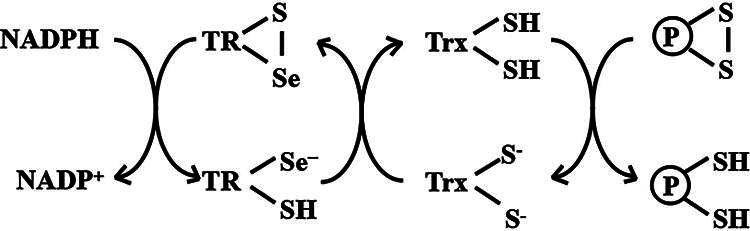
**Redox cycling of the thioredoxin (Trx) system.** The reduced and redox active form of Trx [Trx-(SH)_2_] breaks disulfide bridges between two cysteine residues in oxidized proteins (P–S–S) to generate reduced sulfhydryl proteins [P-(SH)_2_] and oxidized Trx (Trx-S_2_). Trx reductase (TR) recycles Trx-S_2_ into Trx-(SH)_2_. The oxidized TR (TR-S–Se) uses NADPH as electron donors to form catalytically active TR (TR-Se^−^-SH).

The cytosolic Trx system in mammals consists of Trx and Trx reductase-1 (TrxR1). Trx is a small (12 kDa) cell-permeable redox protein with antioxidant and disulfide reductase properties (Das and Das, 2000; Holmgren, 1985; Holmgren and Bjornstedt, 1995). Oxidized Trx (Trx-S_2_) is reduced by TrxR1 utilizing reducing equivalents from NADPH generating reduced Trx (Trx-(SH)_2_ (Fig. 2) (Holmgren, 2000). There are three isoforms of Trx; the most abundant form is the cytosolic Trx and is the focus of this review. In the remainder of this review, it is referred to as Trx. A mitochondrial Trx (Trx-2) (Spyrou et al., 1997) exists, as well as a Trx in spermatozoa (SpTrx) (Miranda-Vizuete et al., 2001). Trxs are evolutionarily highly conserved across the three domains of life, including bacteria, archaea, and eukaryotes (Eklund et al., 1991). The overall 3D structure of all Trxs has a central core of 5 β-strands surrounded by 4 α-helices and the conserved active-site sequence–Cys-Gly-Pro-Cys– (Weichsel et al., 1996). The two Cys residues in the catalytic active site are Cys32 and Cys35 and can form a disulfide bridge when Trx-S_2_, thereby inhibiting its activity (Jeng et al., 1994). Whereas Cys32 and Cys35 perform the direct transfer of electrons to a disulfide, Cys62, Cys69, and Cys73 are involved in regulatory functions in mammalian cells (Haendeler, 2006), and posttranslational modification of these residues such as nitrosylation could alter the activity of Trx (Haendeler, 2006). However, due to the low reduction potential of Trx (*Escherichia coli, E. coli* Trx = –270 mV) (Dyson et al., 1997), Trx is mainly in its reduced form Trx-(SH)_2_ under normal physiological conditions and functions as the major disulfide reductant in the cytosol. We have previously shown that Trx is not a superoxide anion (O_2_^•−^) scavenger (Das and Das, 2000).

**FIG. 3.**
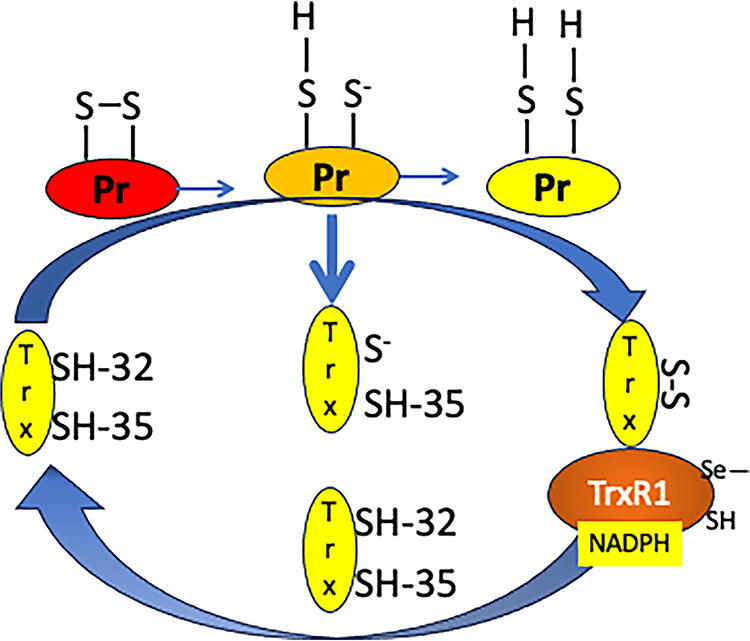
**Sequential catalysis by Cy32 and Cys35 to reduce protein disulfide with regeneration of Trx-SH by TrxR1.** The catalysis occurs in three stages: (1) attack on the target disulfide by active-site nucleophilic cysteine^N^ resulting in transient mixed disulfide between Trx and the target disulfide, (2) formation of intramolecular disulfide bond by attack C-terminal buried cysteine (Cys^C^) residue with reduction of the substrate, and (3) regeneration of the reduced state of Trx by TrxR1 and NADPH (Fig. 3).

Trx specifically converts protein disulfides to reduced thiol containing proteins using TrxR1 and NADPH. The studies by Dyson et al. and Foreman-Kay et al. support the model for Trx redox catalysis by initial attack of the thiolate anion of Cys-32. The Cys residues in the CxxC motif are the active-site Cys32 and the resolving Cys35. The experimentally observed pK_a_ of Cys-32 is 6.3 ± 0.1, while that of Cys-35 is probably between 7.5 and 8.6. These values are in agreement with the previously measured pK_a_ of active-site residues of *E. coli* Trx of 6.4–6.8 and 8.5–9.0 from chemical modification and fluorescence studies and 7.1–7.4 and 8.4 from Nuclear Magnetic Resonance (NMR) analysis (Dyson et al., 1997). The catalysis occurs in three stages as follows: (1) attack on the target disulfide by active-site nucleophilic Cys^N^ resulting in transient mixed disulfide between Trx and the target disulfide, (2) formation of intramolecular disulfide bond by attack of C-terminal buried Cys (Cys^C^) residue with reduction of the substrate, (3) regeneration of the reduced state of Trx by TrxR1 and NADPH (Fig. 3).

Thus, Trx regenerates oxidized proteins to native reduced state. In addition, we have shown that Trx deglutathionylates endothelial nitric oxide synthase (eNOS), which has been shown to be glutathionylated and uncoupled in aging (Kundumani-Sridharan et al., 2021). Trx is a multifunctional protein known to activate transcription factors, induces gene expression, and decreases oxidative stress and inflammation (Holmgren and Bjornstedt, 1995). Trx has been shown to induce its own expression by promoting activation of SP1 transcription factor (Bloomfield et al., 2003). Trx is also secreted in a leaderless pathway (Rubartelli et al., 1992), indicating that intracellular Trx could be secreted out of the cells and could impact various redox-sensitive cell surface receptors. In the cytosol, the redox state is kept in a reduced environment in that Cys residues exist mainly in thiol form rather than in the oxidized disulfide form. Trx is a major disulfide oxidoreductase responsible for maintaining proteins in their reduced state. Although Trx is frequently referred to as an antioxidant, its major function is disulfide reductase activity along with TrxR1 and NADPH, as shown in the above equation. Reduced Trx also provides electrons to Trx peroxidase to reduce H_2_O_2_ to water.

Trx activates proteins *via* an allosteric conformational change. This posttranslational reversible process is comparable with protein phosphorylation and has the potential to alter protein function and activity. Not surprisingly, the Trx system regulates a plethora of physiological processes that involve DNA replication and repair, embryogenesis, inflammation, growth, apoptosis, and angiogenesis that are involved in events ranging from pregnancy (Sahlin et al., 1997), embryonic development (Dong et al., 2016; Jakupoglu et al., 2005; Matsui et al., 1996; Nonn et al., 2003), birth (Das, 2005; Das, 2004; Tipple, 2014), to aging (Altschmied and Haendeler, 2009; Fierro-Gonzalez et al., 2011; Goy et al., 2014; Holmgren and Lu, 2010). During the course of life, it offers protection from cardiovascular diseases (for a review, see Whayne et al., 2015), cancer (Rubartelli et al., 1995), rheumatoid arthritis (Yoshida et al., 1999), AIDS (Nakamura et al., 1996), and more. In this review, we focus on the role of Trx in redox modulation of K^+^ channels. K^+^ channels are linked to many cellular processes, including apoptosis, cardiomyocyte excitation–contraction coupling, vascular hyperpolarization, cellular proliferation, and neuronal stimulation. We are aware that several K^+^ channel types are expressed in one specific cell type and that it is impossible to cover all K^+^ channels in this review. The function of one K^+^ channel may be different in an endothelial cell (EC) compared with a neuron. Hence, we tried to review K^+^ channel redox modulation according to their anatomical position by organizing them for vascular cells, cardiomyocytes, and central nervous cells. The human genome encodes for roughly 200 ion channels. This superfamily of ion channels is involved in many cellular processes, including apoptosis, cardiomyocyte excitation–contraction coupling, vascular hyperpolarization, cellular proliferation, and neuronal stimulation. In this study, we focus on the role of Trx in redox modulation of pore-forming K^+^ channels expressed in vascular cells and cardiomyocytes.

### Small- and intermediate-conductance Ca^2+^-activated K^+^ channels

#### Nomenclature and function

**FIG. 4.**
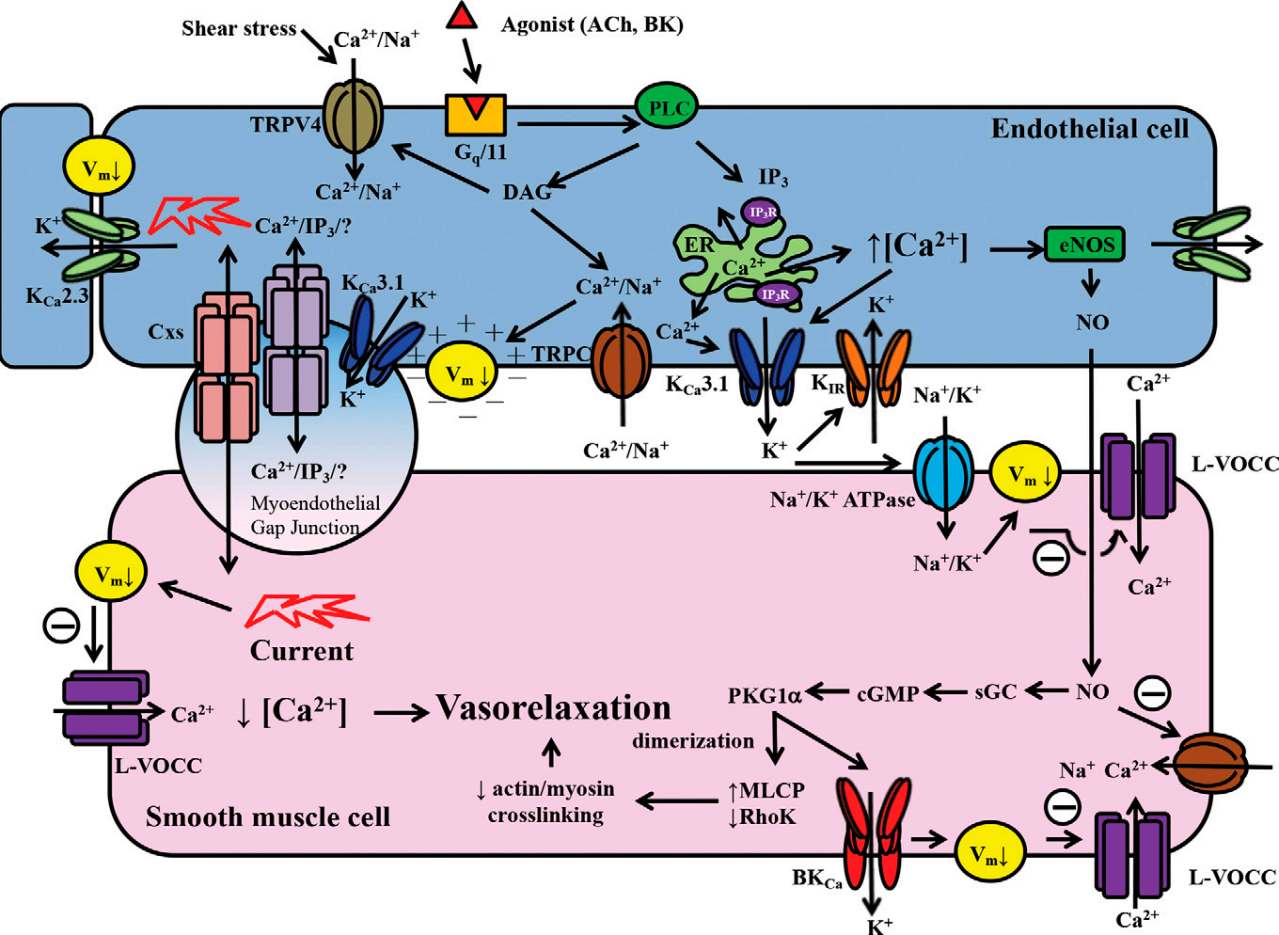
**Schematic overview of the role of vascular ion channels in mediating EC/VSMC communication and hereby regulating vasomotor tone.** The dragging flow of the blood on the endothelial layer creates a shear stress that activates transient receptor potential vanilloid (TRPV) channel leading to an increase in intracellular Ca^2+^ [Ca^2+^]_i_. Agonists such as acetylcholine (ACh) and bradykinin (BK) can bind to G-protein-coupled receptors (G_q_/11) on the EC membrane that couples to phospholipase C (PLC) to activate second messengers such inositol triphosphate (IP_3_) and DAG that result in store-operated Ca^2+^ release. The rise in [Ca^2+^]_i_ activates endothelial Ca^2+^-activated K^+^ channels (K_Ca_2.3 and K_Ca_3.1) to trigger K^+^ efflux that hyperpolarizes the EC membrane. This hyperpolarization current can spread *via* other K_Ca_2.3 channels to neighboring ECs. The current can spread through connexin channels located in myoendothelial gap junctions. The extracellular K^+^ activates inward-rectifier K^+^ channels (K_IR_) and Na^+^/K^+^ ATPase located on the VSMC membrane resulting in a decrease in membrane potential (↓ Vm) and subsequent VSMC hyperpolarization. Hyperpolarization inhibits L-VOCC, preventing VSMC Ca^2+^ increase suppressing contraction and causing vasorelaxation. In addition, a rise in EC [Ca^2+^]_i_ activates endothelial nitric oxide synthase (eNOS) to generate NO, which diffuses to the VSMC to activate soluble guanylate cyclase (sGC). This generates cyclic guanidine monophosphate (cGMP), a second messenger known to stimulate protein kinase G (PKG1α). PKG1α can activate large-conductance Ca^2+^-activated K^+^ channels (BK_Ca_), increase myosin light chain phosphatase (MLCP) activity, and decrease Rho kinase activity, reducing actin/myosin crosslinking and contraction. EC, endothelial cell; VSMC, vascular smooth muscle cell.

Calcium-activated K^+^ (K_Ca_) channels open in response to increases in intracellular [Ca^2+^] and contribute to the afterhyperpolarization in neurons and cardiomyocytes, and also in nonexcitable cells such as lymphocytes, erythrocytes, fibroblasts, secretory epithelial, and vascular ECs (Adelman et al., 2012; Grgic et al., 2009; King et al., 2015; Wulff and Castle, 2010; Zhang et al., 2015). In the vasculature, many K^+^ channels are involved in regulating vasomotor tone. K_Ca_ channels of different conductance have been identified, including small (10–15 pS), intermediate (20–60 pS), and large (200–300 pS) conductance channels. Three highly conserved small-conductance K_Ca_ (SK) channels have been cloned from the rat and human brain (SK1, SK2, and SK3), each containing 6 putative transmembrane segments (Kohler et al., 1996). A vascular apamin-sensitive SK3 channel has been cloned as well (Sokol et al., 1994), which has now been confirmed to be expressed in the endothelium (Burnham et al., 2002). This endothelial SK3 channel is also named K_Ca_2.3. A fourth SK subtype (SK4) is now classified as the intermediate-conductance K_Ca_ (IK_Ca_) channel, also referred to as IK1, but preferably as K_Ca_3.1 (Ishii et al., 1997; Joiner et al., 1997). Endothelial K_Ca_2.3 and K_Ca_3.1 channels are voltage-insensitive and demonstrate a nonohmic current/voltage relationship with a conductance two to three times higher for inward than outward currents (Xia et al., 1998). Hence, they belong to the family of inward-rectifier K^+^ (K_IR_) channels (see the Inward-Rectifier K^+^ Channels section). Channel opening results in K^+^ efflux, which hyperpolarizes the EC membrane. This hyperpolarizing current is spread through adjacent ECs (*via* K_Ca_2.3) (Lin et al., 2012) and to vascular smooth muscle cells (VSMCs) *via* myoendothelial junctions (*via* K_Ca_3.1) (Dora et al., 2008; Sandow et al., 2006) or *via* activation of Na^+^/K^+^-ATPase and K_IR_ channels (Edwards et al., 1998) resulting in VSMC hyperpolarization and subsequent relaxation (Fig. 4). This process is called endothelium-dependent hyperpolarization (EDH). Mice deficient in K_Ca_2.3 and/or K_Ca_3.1 display a disrupted EDH response and are hypertensive (Brahler et al., 2009; Si et al., 2006; Yap et al., 2016). Angiotensin II (Ang II)-induced hypertension has been shown to reduce the expression of these channels in rat mesenteric arteries (Hilgers et al., 2006). Besides its role in blood pressure regulation, the K_Ca_3.1 channel modulates ion transport in epithelial cells (Singh et al., 2001), renal fibroblast proliferation and fibrogenesis (Grgic et al., 2009), and is a key cellular effector in several life-threatening diseases such as atherosclerosis, restenosis, and traumatic injury-induced edema (Kohler et al., 2003; Tharp and Bowles, 2009; Toyama et al., 2008). Knowledge in the molecular and functional understanding of K_Ca_ channel regulation is vital for the design of putative therapeutic K_Ca_ channel gating modulators applicable to a wide variety of health disorders (Christophersen and Wulff, 2015; Kohler et al., 2016).

#### Structure

All K^+^ channels have essentially the same pore constitution, containing a critical amino acid sequence, the K^+^ channel signature sequence or regulator of conductance of K^+^ (RCK) domain (Heginbotham et al., 1994; Jiang et al., 2001; Kuang et al., 2015; Pau et al., 2011). This sequence is a part of the selectivity filter. The endothelial K_Ca_ channel is a tetrameric protein with each subunit compromising 427 amino acids organized in six transmembrane (S1-S6) segments. The first structural data for pore formation of K^+^ channels came with the solved crystal structure of the bacterial K^+^ channel KcsA (Doyle et al., 1998). The K^+^ channel resembles an inverted “teepee-like” structure in that the four inner helices pack against each other similar to a flower bundle near the cytosolic side of the membrane (Doyle et al., 1998). The pore helices are slotted in between and are pointed near the center of the channel. Herein lays the selectivity filter that binds and conducts K^+^ ions. This pore region in S5-S6 contains a conserved Glycine-Tyrosine-Glycine (GYG) pore motif (Lipkind et al., 1995).

#### Activity and redox modulation

Sensitivity to Ca^2+^ arises from binding of Ca^2+^ to calmodulin (CaM) (Khanna et al., 1999; Xia et al., 1998). This CaM binding domain (CaMBD) for the K_Ca_3.1 channel is located at the C-terminal end of the S6 segment (residues Lys312-Thr329) (Morales et al., 2013), whereas in the K_Ca_2.3 channel, the CaMBD resides deeper in the channel pore in or near the selectivity filter (Bruening-Wright et al., 2002). Upon binding of Ca^2+^ to CaM, the CaM/CaMBD complex undergoes a conformational change, which is coupled to the channel pore enabling the channel to transition from a nonconducting to a conducting configuration (Fig. 4) (Jiang et al., 2002; Keen et al., 1999; Li et al., 2009; Schumacher et al., 2001). The S6 segment proximal to the GYG motif extending from Val266 to Ala286 is preserved in K_Ca_3.1, rat SK2, the bacterial K^+^ channel KcsA, and voltage-gated Shaker K^+^ channels (Simoes et al., 2002). The S5-pore-S6 region is most likely to play a crucial role in the K_Ca_3.1 pore formation and channel gating. Simoes and colleagues used a technique called substituted cysteine accessibility mutagenesis (SCAM) (Akabas, 2015; Akabas et al., 1992). All residues between Val275 and Ala286 were mutated to Cys residues and these K_Ca_3.1 mutants were expressed in *Xenopus laevis* oocytes (Simoes et al., 2002). Next the effects of the water-soluble methanethiosulfonate (MTS) derivates sodium (2-sulfonatoethyl) methanethiosulfonate (MTSES), 2-aminoethyl methanethiosulfonate hydrobromide (MTSEA), and [2-(trimethylammonium) ethyl] methanethiosulfonate bromide (MTSET) on the single-channel conductance, and gating properties of Cys K_Ca_3.1 mutants were examined. These thiol-reactive agents reversibly block Cys residues and other sulfhydryl groups (–SH into –S-S-CH_2_-CH_2_-SO_3_^–^ in the case of MTSES, –S-S-CH_2_-CH_2_-NH_3_^+^ in the case of MTSEA, and –S-CH_2_-CH-N-(CH_3_)^3+^ in the case of MTSET). Covalent addition of the side group to the S-atom of Cys may interfere with ion permeation if the modified side chain is positioned in or near the ion conduction pathway. Hence, these thiol-reactive agents provide important information on the location of Cys residues in the channel, since these water-soluble agents specifically bind to Cys residues that are accessible *via* the pore or are located near the cytoplasmic C-terminus. Simoes and colleagues observed that interaction of MTSET with these Cys residues leads to current inhibition, and closure by the gate occurs at a point between the luminal Thr278 and Val282 residues (Simoes et al., 2002). Further studies by this group showed that in closed conformation a narrow passage is created centered at Val282 that connects the channel cavity to the cytosol. However, this passage (∼10 Å) is not narrow enough to restrict passage of small reagents such as MTSEA but forms a tight seal permeable to smaller reagents and K^+^ ions (Garneau et al., 2009; Klein et al., 2007). Channel activation should occur distal to Val282 at the cytoplasmic COOH terminus (Ala283 and Ala286) (Garneau et al., 2009; Klein et al., 2007). However, in contrast to the previous investigations, which determined the role of Cys engineered along the luminal face of S6, the nonluminal face of S6 also participates in the activation mechanism. Interestingly, this nonluminal S6 region contains 2 adjacent Cys residues (Cys 276 and −277), which have the potential to undergo posttranslational modifications (Klein et al., 2007). Cys modulation with the thiol-reactive and membrane-impermeable agent parachloromercuribenze sulfonate (PCMBS) at Cys276 shifts the gating equilibrium toward the open conformation (Bailey et al., 2010). Substituting the hydrophobic Val282 with hydrophilic amino acids locks the channel in an open-like state, resulting in channels that are ion conducting in the absence of Ca^2+^ (Garneau et al., 2010). Hence, the nonluminal face of S6 forms a critical interaction surface, which shifts to the closed conformation. Thus, the luminal and nonluminal C-terminal portion of S6 could be allosterically coupled to the activation gate. H_2_O_2_ and other thiol reactive agents such as 5,5′-dithio-bis (2-nitrobenzoic acid) (DTNB or Ellman’s reagent) or [(O-carboxyphenyl)thio]ethyl mercury sodium salt (thimerosal) were shown to inhibit K_Ca_3.1 channel activation in bovine aortic ECs only when applied on the intracellular side (Cai and Sauve, 1997). Channel activity could partly be restored by disulfide reducing agents dithiothreitol (DTT) or reduced glutathione. However, there are limited studies that determined Cys redox modulation of endothelial K_Ca_ channels in *ex vivo* models *via* EDH response in isolated ECs or contracted arteries. Hyperhomocysteinemia (HHCy) has been shown to inhibit the EDH response in murine small mesenteric arteries *via* oxidative and nitrosative stress, which was inhibited by catalase and the peroxynitrite inhibitor ebselen (Cheng et al., 2011). Auto-oxidation of HCy produces H_2_O_2_ resulting in conversion of HCy into its disulfides, which is mediated by thiol–disulfide exchange reactions (Sengupta et al., 2001). Upregulation of Trx suppresses HCy-induced reactive oxygen and nitrogen species (RONS) formation (Dai et al., 2008). In patients with combined coronary artery disease and HHCy, serum Trx levels are decreased (Wu et al., 2010). Taken together, a protective role for Trx in preventing and reducing disulfides and endothelial dysfunction associated with HHCy is likely, although more studies are needed to be performed in this area.

**FIG. 5.**
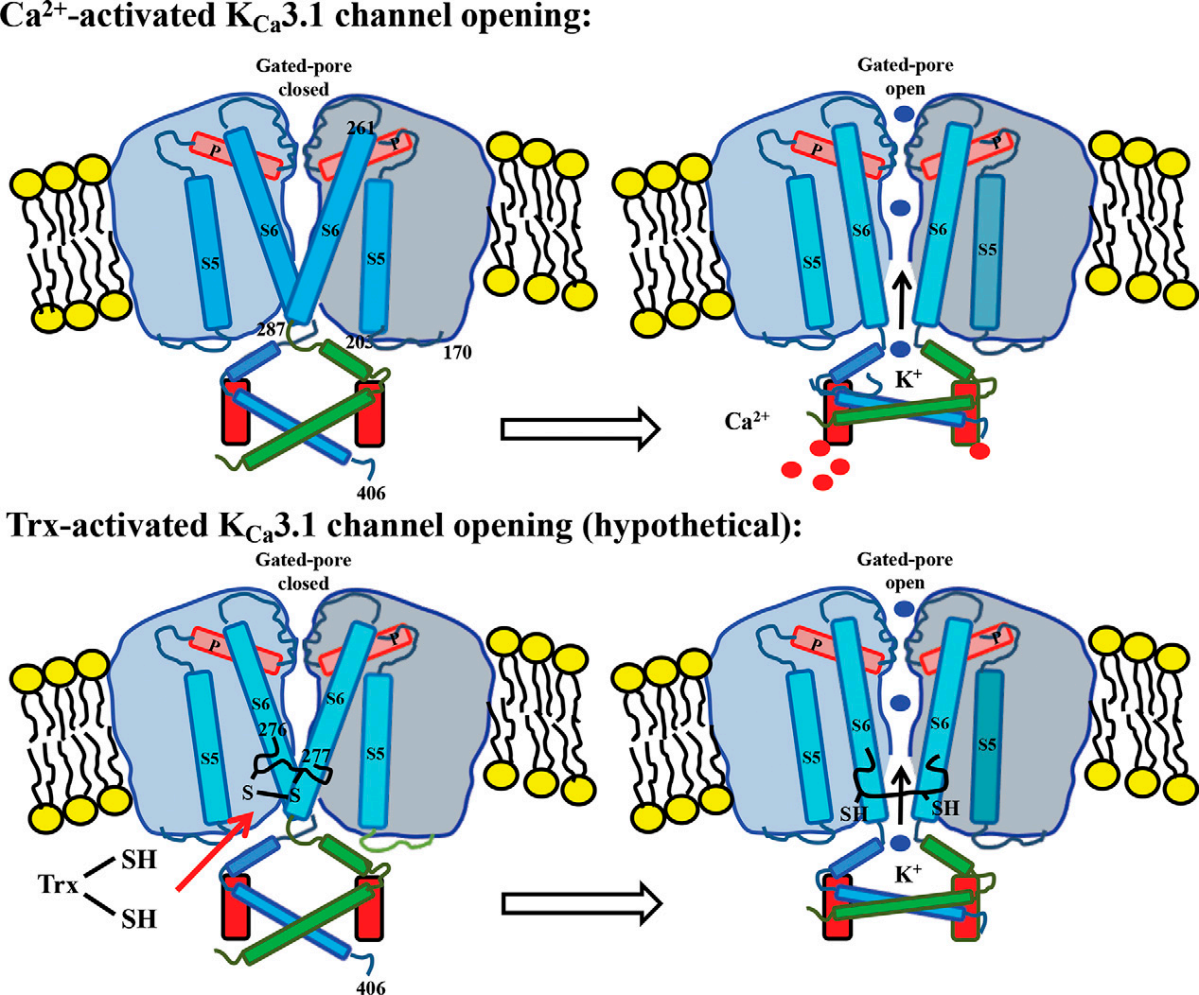
**Mechanism of Ca^2+^-activated and Trx-activated K_Ca_3.1 channel opening.** Binding of Ca^2+^ to the calmodulin-binding domain located at the intracellular side of the K_Ca_3.1 channel results in a conformational change opening the channel gate to allow passage of K^+^ ions (upper figure). A hypothetical model of K_Ca_3.1 is presented in the lower figure in which active and reduced thioredoxin [Trx-(SH)_2_] breaks a disulfide cysteine bridge between Cys-276 and −277 resulting in a conformational change opening the channel gate. Cys, cysteine; Trx, thioredoxin.

Recently, we have demonstrated that the K_Ca_3.1 component of this EDH response was enhanced in *ex vivo* small mesenteric arteries from mice overexpressing human Trx (Trx-Tg) (Hilgers and Das, 2015) and that the oxidizing agent diamide inhibited the EDH, which was reversed by DTT, suggesting a redox regulation of the EDH response (Hilgers and Das, 2015). Furthermore, mice expressing a mutant redox inactive Trx (dnTrx-Tg) had severely blunted EDH response (Hilgers and Das, 2015). This suggests that the EDH response is redox regulated. Although our study demonstrated redox control of EDH by Trx, a direct mechanistic link between Trx and K_Ca_3.1 channel Cys modulation needs to be further studied and confirmed (Fig. 5).

### Inward-rectifier K^+^ channels

#### Nomenclature and function

The inward-rectifier K^+^ channel (K_IR_) activation results in hyperpolarization and VSMC relaxation in a number of vascular beds, including coronary (Knot et al., 1996), cerebral (Knot et al., 1996; Quayle et al., 1993), cremaster (Loeb et al., 2000), mesenteric (Dora et al., 2000), and renal arteries (Chilton and Loutzenhiser, 2001). The K_IR_2.1 subtype is expressed in ECs and mediates EDH together with K_Ca_2.3 and K_Ca_3.1 channels (Sonkusare et al., 2016). K_IR_ channels are unique among K^+^ channels in that their conductance is increased by a small rise (to <20 mM) in the extracellular K^+^ concentration. These channels differ from other K^+^ channels where small amounts of extracellular K^+^ (<20 mM) increase K_IR_ current leading to hyperpolarization. K_IR_ channels can be further separated on the basis of their functional characteristics: classical, G-protein gated, ATP-sensitive (K_ATP_), and K^+^ transport channels (Hibino et al., 2010). K_IR_2.1 and K_IR_2.2 are expressed in VSMCs from cerebral arteries where they mediate parenchymal arteriolar vasodilation (Longden et al., 2014; Wu et al., 2007). ATP inhibits K_ATP_ channels at micromolar intracellular concentrations in pancreatic β cells resulting in insulin release, whereas endogenous substances such as adenosine, neuropeptides, and endothelial factors open K_ATP_ channels resulting in vasorelaxation (Babenko et al., 1998; Quayle et al., 1997).

#### Structure

The K_IR_ channel family consists of seven subfamilies (K_IR_1–7) comprising a total of 15 subunit isoforms (K_IR_1.1, K_IR_2.1–4, K_IR_3.1–4, K_IR_4.1–2, K_IR_5.1, K_IR_6.1–2, and K_IR_7.1). Each subunit is a two-transmembrane protein connected by a pore-forming loop with intracellular C- and N-terminal domains. Functional K_IR_ channels are tetramers formed from homo- or heteromeric assemblies of individual subunits. K_ATP_ channels consist of four K_IR_ subunits (K_IR_6.1 and K_IR_6.2). Each subunit is associated with a larger sulfonylurea receptor (SUR) subunit assembled into an octameric protein (Brayden, 2002). The combination K_IR_6.2/SUR2B is likely the most prevalent in VSMCs. The primary structure of the human K_IR_2.1 channel contains a total of 13 Cys residues, most of which appear to be cytosolic, with two external Cys at positions 122 and 154 highly conserved among K_IR_ channels. Cys122 and Cys154 in the K_IR_2.1 channel cause disulfide bond formation, which is required for correct folding of KIR2.1 (Cho et al., 2000; Leyland et al., 1999).

#### Activity and redox modulation

**FIG. 6.**
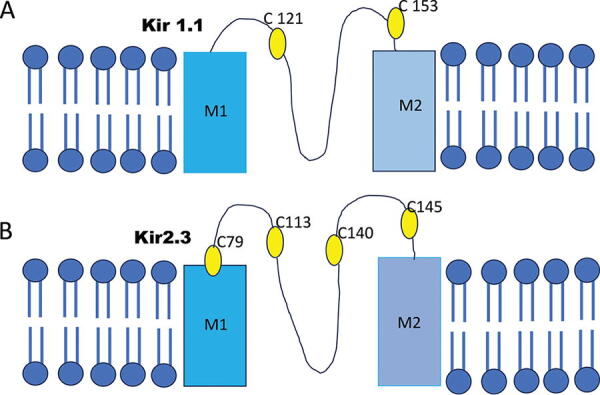
**External cysteines in Kir1.1 and Kir2.3 channels modulate channels intrinsic kinetic properties and also gating properties.** Wild-type Kir 2.3 currents were significantly inhibited by PCMBS, thimerosal, and H_2_O_2_. Currents for mutant Kir2.3 C79S and C140S were also inhibited by PCMBS, thimerasol, and H_2_O_2_. These mutations affected the time-course of inhibition by all three agents. The reducing agent DTT reversed the inhibition by both PCMBS and thimerosal of wild-type and mutant currents. These cysteines also contribute to the gating properties of the Kir2.1 channel. H_2_O_2_, hydrogen peroxide; DTT, dithithreitol; PCMBS, Parachoromercuribenzene sulphonate.

SCAM experiments have shown that Cys49 and Cys308 in K_IR_1.1, responsible for K^+^ secretion in the kidney, are susceptible to thiol oxidation by DTNB or MTSES, which results in irreversible channel closing (Schulte et al., 1998). Using a similar approach, Cys54 and Cys76 in K_IR_2.1 are modified by MTSET and it was proposed that these Cys residues could participate in the formation of an inner vestibule facing the channel central pore (Lu et al., 1999). Substitution of Cys76 by a polar residue (Ser) reduced channel activity and the open probability (Garneau et al., 2003). Mutation of the C-terminal Cys311 to Ser311 also decreased channel activity (Garneau et al., 2003). Interestingly NO activates cardiac K_IR_2.1 at Cys76 *via* S-nitrosylation (Gomez et al., 2009). Cys311 is important in drug-induced K_IR_2.1 channel opening (Gomez et al., 2014). Bychkov and colleagues have demonstrated a differential effect of H_2_O_2_ on endothelial K^+^ channel activation. Low concentrations of H_2_O_2_ (0.01–0.25 µM) inhibited K_IR_ channel current resulting in EC depolarization, whereas high concentrations of H_2_O_2_ (>500 µM) activated the K_Ca_ current resulting in hyperpolarization (Bharadwaj and Prasad, 1995; Bychkov et al., 1999). The heteromeric K_IR_4.1/K_IR_5.1 channel is expressed in the kidney, brain stem, and retina. Exposure to H_2_O_2_ or diamide resulted in channel inactivation, whereas Cys158 in the K_IR_5.1 channel was S-glutathionylated by oxidized glutathione (GSSG) and inhibited the channel (Jin et al., 2012). The authors postulate that the GSH moiety occupies the ion-conducting pathway leading to channel inhibition. In a classical experiment, Bannister et al. used the Cys reactive agent, PCMBS, thimerosal, and H_2_O_2_ to inhibit Kir2.3 channel and compared the results with that of Kir1.1 channel (Fig. 6). They also made mutation of Cys to serine by site-directed mutagenesis (Bannister et al., 1999). Wild-type Kir 2.3 currents were significantly inhibited by PCMBS, thimerosal, and H_2_O_2_. Currents for mutant Kir2.3 C79S and C140S were also inhibited by PCMBS, thimerasol, and H_2_O_2_. These mutations affected the time-course of inhibition by all three agents (Bannister et al., 1999). The reducing agent DTT reversed the inhibition by both PCMBS and thimerosal of wild-type and mutant currents, but not the inhibition by H_2_O_2_. Thus, these results showed that inhibition of Kir2.3 channel is mediated by C79, a slow component, and by C140, a faster component, both residues are externally exposed (Bannister et al., 1999). Studies by Garneau et al. demonstrated that substitution of N-terminal Cys C76 or the C-terminal Cys C311 by polar residues strongly modifies the channels’ intrinsic kinetic properties with the mutation position 311, introducing long-lasting closed time intervals through a destabilization of the channel PIP2-Kir2.1 interaction (Garneau et al., 2003). Kir 2.1 model predicts that seven of the 13 Cys residues of the channel are distributed along the N- and C-terminus region with some of the residues comprised within highly conserved domains involved in channel gating (Garneau et al., 2003). Thus, these Cys contribute to the gating properties of the Kir2.1 channel (Garneau et al., 2003).

Previous studies have shown that K_ATP_ channels are inhibited by oxidative stress resulting in impairment in vasorelaxation (Armstead, 1999; Miura et al., 2003). However, ONOO^−^ hyperpolarizes and results in relaxation of the rabbit internal carotid artery through activation of K_ATP_ channels (Ohashi et al., 2005). Sulfhydryl oxidizing agents such as diamide, GSSG, and micromolar concentrations of H_2_O_2_ have been shown to inhibit the K_IR_6.2/SUR2B channel (Yang et al., 2010). Oxidant screening of Kir6.1-Kir6.2 chimeras demonstrated that the N terminus and transmembrane domains of Kir6.1 were crucial (Yang et al., 2011). Systematic mutational analysis revealed three Cys residues in these domains: Cys43, Cys120, and Cys176 (Yang et al., 2011). This oxidant-mediated channel inhibition was rescued by DTT and the glutaredoxin, suggesting S-glutathionylation. In contrast to Cys176, Cys43 had only a modest contribution to S-glutathionylation, and Cys120 was modulated by extracellular oxidants but not intracellular GSSG. Using systematic Cys residue mutation and simulation modeling, the authors demonstrated that after binding to Cys176, the GSH moiety occupies a space between the slide helix and the transmembrane helices (Yang et al., 2011). Functional gating of the K_ATP_ channel requires a conformational change of these transmembrane helices for channel opening, which is hindered by the GSH moiety retaining the K_IR_6.1 channel in a closed state (Yang et al., 2011). Mutation of Cys166 of K_IR_6.2 situated at the cytosolic end of S2, increases the open probability consistent with the idea that Cys166 plays a role in the intrinsic gating of the channel, possibly by influencing a gate located at the intracellular end of the pore (Trapp et al., 1998). These reports evidently show that oxidative stress results in Cys sulfhydryl oxidation and K_ATP_ channel inhibition, which can lead to impairment of vasorelaxation in a variety of vascular complications. H_2_S causes Cys S-sulfhydration (Mustafa et al., 2009) and subsequent activation of K_IR_6.1/SUR2B channels in piglet cerebral arterioles (Jiang et al., 2010). Trx has recently been shown to cleave Cys S-sulfhydration resulting in H_2_S release (Mikami et al., 2011; Wedmann et al., 2016). Based on these reports, it is likely that Trx could play a critical role in channel activation or inhibition depending upon the Cys residue oxidation state and the accessibility of the Cys residues for reduction by Trx. However, the role of Trx is largely unexplored in these channel functions. We have recently shown that Trx deglutathionylates glutathionylated eNOS (SG-eNOS) (Subramani et al., 2021). Based on this study, Trx could also deglutathionylate K^+^ channel Cys glutathionylated in oxidative stress conditions, thereby increasing or decreasing channel function. The role of Trx is largely unexplored in the K^+^ channel function.

### Large-conductance K_Ca_ channels

#### Nomenclature and function

Large-conductance Ca^2+^-activated K^+^ channels (BK_Ca_), also known as Slo or Maxi-K channels, play important roles in oxygen sensing, VSMC relaxation, synaptic transmission, and hormone secretion (Hou et al., 2009; Nelson and Quayle, 1995). BK_Ca_ mediate the voltage-dependent and Ca^2+^-dependent K^+^ permeability involved in VSMC K^+^ efflux and hyperpolarization. One BK_Ca_ channel complex contains four pore-forming α subunits (BKα, KCNMA1, *Slowpoke*, Slo or Slo1) and up to four regulatory β subunits (BKβ, KCNMB1-4) (Brenner et al., 2000a; Knaus et al., 1994; Toro et al., 1998; Wang et al., 2002). Each α subunit includes a seven-helix transmembrane segment and a voltage-sensing domain (S1–S4) and contributes one-fourth of the ion conduction pore (S5–S6)(Adelman et al., 1992). β subunits consisting of 2 transmembrane units are involved in voltage sensitivity, BK_Ca_ channel inhibition *via* the “ball peptide” mechanism (see the Voltage-Dependent K^+^ Channels section) (Bentrop et al., 2001; Xia et al., 2003), and pharmacological inhibition by peptide blockers such as charybdotoxin and iberiotoxin (Meera et al., 2000). At the C-terminal end of the BK_Ca_ channel (past S6), there is a large cytoplasmic region, which contains two RCK domains (regulator of K^+^ conductance RCK1 and RCK2) essential for Ca^2+^ activation of the channel (Xia et al., 2002). RCK1 also serves as an H^+^ sensor (Hou et al., 2008). A segment linking both RCKs has been implicated in heme binding (Horrigan et al., 2005; Tang et al., 2003). A genetic disruption of the BK β1 subunit impairs vascular relaxation and causes chronic hypertension by inhibiting the Ca^2+^-dependent activation of BK_Ca_ channels (Brenner et al., 2000b; Pluger et al., 2000). During Ang II-induced hypertension, expression of the β_1_ subunit of the BK_Ca_ protein is downregulated in rat cerebral arteries (Amberg and Santana, 2003). The expression of the β_1_ subunit of BK_Ca_ is downregulated in cerebral artery VSMCs from diabetic rats resulting in decreased BK_Ca_ activity and hypertension (Wang et al., 2010). These observations highlight the importance of vascular BK_Ca_ channels in regulating vasomotor tone and blood pressure.

#### Activity and redox modulation

Physiologically, BK_Ca_ channels are activated, other than membrane potential (V_m_) and Ca^2+^, by cGMP-dependent phosphorylation (Carrier et al., 1997), H_2_S (Jackson-Weaver et al., 2011), and RONS (Wang et al., 1997b). In the carotid body, the BK_Ca_ channel is sensitive to O_2_ concentration (Lewis et al., 2002; Liu et al., 1999). As described above, the BK_Ca_ channel contains a heme binding domain. Heme binding strongly inhibits the channel under hypoxic conditions, whereas under normoxic conditions, carbon monoxide (CO), which is generated by heme oxygenase-2 (HO-2) during heme degradation, activates the BK_Ca_ channel (Jaggar et al., 2005). This heme binding domain contains a Cys612-X-X-Cys615-His motif that serves as the heme ligand provided by His616. This dithiol binds to HO-2 with 14-fold more tightly than the oxidized disulfide state (Yi et al., 2010; Yi et al., 2009). Under hypoxic (reduced redox potential) conditions, the BK_Ca_ channel converts to the dithiol state having high affinity for Fe^3+^-heme, which inhibits BK_Ca_ channel leading to pulmonary vasoconstriction. Under normoxia, the situation is reversed: the BK_Ca_ channel is in its oxidized disulfide state having a high affinity for CO, which activates BK_Ca_ activity. In addition, Barnes et al. (2018) have shown that mice with deletion of Kcnmb1 gene (Kcnmb1^−/−^) that encodes for β1 subunit of BK_Ca_ channel showed increased right ventricular systolic pressure compared with Kcnmb1^+/+^ mice when exposed to chronic or acute hypoxia. β1 subunit expression was predominantly confined to pulmonary artery smooth muscle cells (PASMCs). Compromised PASMC β1 function may contribute to heightened microvascular vasoconstriction that characterizes pulmonary hypertension.

The mechanism of oxidative stress-induced BK_Ca_ channel modulation is complex and many studies have observed conflicting outcomes whether oxidation increases or decreases BK_Ca_ channel activity (for an extensive review, see Hermann et al., 2015). The inconsistency might arise from different experimental approaches (*e.g.,* isolated cells using patch clamp *versus* intact precontracted vessels, intra-*versus* extracellular exposure, and concentration of oxidative agents applied), species/cell differences, or multiple Cys modulation sites having different gating effects (Sandow and Grayson, 2009). Numerous studies have observed BK_Ca_ channel activation by exogenously applied H_2_O_2_ (Barlow et al., 2000; Barlow and White, 1998; Bychkov et al., 1999; Fraile et al., 1994; Hayabuchi et al., 1998; Iida and Katusic, 2000; Matoba et al., 2002; Matoba et al., 2000; Thengchaisri and Kuo, 2003; Wei et al., 1996; Yang et al., 1998). Arachidonic acid (AA) triggers endogenous H_2_O_2_ release that activates BK_Ca_ channel causing *in vivo* cerebral arteriolar vasodilatation (Paravicini et al., 2004; Sobey et al., 1997; Sobey et al., 1998). Barlow and colleagues have shown that H_2_O_2_ relaxes coronary arteries by BK_Ca_ channel activation *via* the phospholipase A_2_/AA signaling cascade (Barlow et al., 2000). Furthermore, overexpression of glutathione peroxidase-1 and knockout of SOD1 blunted AA-induced cerebral arteriolar dilations (Modrick et al., 2009), indicating that endogenously produced H_2_O_2_ also activates BK_Ca_ channels. H_2_O_2_ activates soluble guanylate cyclase resulting in guanosine 3′−5′-cyclic monophosphate (cGMP) release in the pulmonary vasculature (Burke-Wolin et al., 1991). Protein kinase G (PKG) is activated by cGMP, and protein kinase G alpha (PKG1α) is an important regulator of various K^+^ channels, including BK_Ca_ channel (Schubert and Nelson, 2001). Opening of BK_Cs_ channel requires a signaling involving H_2_O_2_-mediated dimerization and translocation of PKG1α (Zhang et al., 2012). Zhang and colleagues provided compelling evidence that H_2_O_2_ relaxes human coronary arteries *via* PKG1α Cys oxidation and opening of BK_Ca_ channels (Zhang et al., 2012). In addition, PKG1α induces a decrease in VSMC Ca^2+^ and inhibits Rho kinase activity resulting in increased myosin light chain phosphatase activity *via* binding to its myosin phosphatase target subunit 1 (MYPT1) hereby reducing myosin actin crossbridge cycling and subsequent relaxation (Hofmann et al., 2000). It is now evident that PKG1α is redox sensitive at Cys42 because Cys thiol oxidation causes a disulfide bond formation between two adjacent Cys42 residues of PKG creating a catalytically active dimer (Burgoyne et al., 2007). This homodimer complex is independent of cGMP and has a higher affinity for substrate. cGMP binding to PKG1α results in an allosteric conformation that reorientates redox Cys residues and prevents or attenuates dimerization (Busch et al., 2002; Schnell et al., 2005). The importance of this Cys42 thiol oxidation in PKG has been confirmed by a single-atom mutation (oxygen instead of sulfur) in Cys42. Generation of a Cys42Ser PKG1α knockin mouse eliminated oxidant sensing (Prysyazhna et al., 2012). Hence, autoregulation of cGMP and the resulting reorientation move the Cys42 too far apart rendering PKG1α relatively more resistant to oxidant-induced disulfide formation (Burgoyne et al., 2012). A recent study has also shown that the constitutive activation of PKG Iα proceeds through oxidation of Cys-117 (Sheehe et al., 2018).

In contrast to these vasorelaxing effects of H_2_O_2_-induced BK_Ca_ channel stimulation, studies show that the activity of BK_Ca_ can be inhibited by H_2_O_2_ (Brakemeier et al., 2003; DiChiara and Reinhart, 1997; Lu et al., 2006; Soto et al., 2002; Tang et al., 2001). Equally conflicting observations have been reported studying the exogenous effects of ONOO^−^, which has been either shown to inhibit vascular BK_Ca_ in cerebral and coronary VSMCs (Brzezinska et al., 2000; DeWitt et al., 2001; Elliott et al., 1998; Liu et al., 2002; Tang et al., 2004) or activate K^+^ channels (Li et al., 2004; Ohashi et al., 2005). Using patch-clamp techniques in *Xenopus laevis* oocytes expressing BK_Ca_ channels, exposure of H_2_O_2_ to the intracellular side resulted in channel inactivation, whereas extracellular application had no effect (Soto et al., 2002). This suggests an intracellular inhibition site. Extracellularly applied H_2_O_2_ causes Ca^2+^ leakage due to membrane damage, resulting in a nonspecific rise in intracellular Ca^2+^, which activates BK_Ca_ channels (Gupta et al., 1998; Whyte et al., 2009). Taken together, Cys thiol modifying agents such as DTNB and MTSEA inhibit the BK_Ca_ current (DiChiara and Reinhart, 1997; Soh et al., 2001; Soto et al., 2002; Tang et al., 2001; Wang et al., 1997b), suggesting that oxidative modification of Cys residues in the BK_Ca_ channel promotes inhibition of BK_Ca_ channel function. This has been confirmed by using a mutant BK_Ca_ protein, in which Cys911 of the human slo1 (BKα) subunit is changed into Ala911 (Tang et al., 2004). Cys911 is in the Ca^2+^ bowl mediating the Ca^2+^-sensitivity of the slo1 channel (Niu and Magleby, 2002; Xia et al., 2002). Cys911 is elegantly shown to mediate the oxidation sensitization of the hslo1 channel during physiological Ca^2+^ concentrations. Absence of a free thiol group at Cys911 disrupts the Ca^2+^ bowl function by interfering with the Ca^2+^-binding step and/or with coupling of the Ca^2+^ sensor and the channel gate. In addition to Cys911, modification of Cys430 in the cytoplasmic regulator of potassium conductance domain alters the Ca^2+^-dependent activation of Slo1 (Zhang and Horrigan, 2005; Zhang et al., 2006). Hyperglycemia results in enhanced endogenous H_2_O_2_ production and BK_Ca_ channel inhibition *via* Cys911 oxidation (Lu et al., 2006). This implies that endogenously produced H_2_O_2_ can inhibit BK_Ca_ channel activity *via* Cys thiol oxidation. However, predicting whether oxidation will result in BK_Ca_ channel stimulation or inhibition is difficult as evidenced by the above studies. For instance, methionine oxidation by chloramine T increases BK_Ca_ channel activity, whereas Cys oxidation decreases BK_Ca_ channel activity (Tang et al., 2001). Even in the same cell type, pyramidal neurons from the hippocampal CA1 region, opposing effects of oxidizing agents (GSSG and DTNB) have been observed on BK_Ca_ channel activation (Gao and Fung, 2002; Gong et al., 2000; Soh et al., 2001). Nonetheless, it can be concluded that BK_Ca_ channel activity is highly sensitive to Cys thiol modulation. Modulation of different Cys residues in the BK_Ca_ channel may result in pore opening or gate closing depending on the location of that Cys residue. Recently, Hollywood et al. have shown that leucine-rich repeat and Ig domain containing 2 (LINGO2) is a regulator of BK channels, since its coexpression with BK channels yields rapid inactivating currents, the activation of which is shifted approximately −30 mV compared with that of BKα currents. Furthermore, they show that oxidation of BK: LINGO2 currents (by exposure to epifluorescence illumination or chloramine T) abolished inactivation. The effect of illumination was dependent on the presence of green fluorescent protein, suggesting that it released free radicals, which could have oxidized Cys or methionine residues. In addition, the oxidation effects were resistant to treatment with the Cys-specific reducing agent DTT, suggesting that methionine rather than Cys residues may be involved (Dudem et al., 2023). Therefore, we speculate that Trx may alter the redox state of LINGO2 or BK due to its reducing activity and could play a significant role in Cys disulfide reduction in these Cys residues present on BK_Ca_ channels or other regulators for these channels. Although the role of biological reductant such as Trx has not been studied in altered BK_Ca_, we hypothesize a significant role of Trx in the redox control of BK_Ca_. Although Trx can reduce oxidized Cys in general, accessibility of oxidized bonds is a critical issue for reduction by Trx, as it is a 12kD protein and needs to have physical contact with the oxidized protein to be able to exchange electrons. It cannot reduce buried Cys disulfides in the folded proteins.

#### Heterogeneity in vasorelaxation between conduit and resistance arteries

Based on the above differential effects of Cys modulation on K_Ca_ channels, it is not surprising that not all arteries function similarly in response to vasoactive compounds. For example, heterogeneity in the relaxation of different sized porcine coronary arteries to nitro-vasodilators has been observed. NO and nitro-vasodilators cause vasodilatation *via* phosphorylation of PKG- and cGMP-mediated BK_Ca_ activation (Alioua et al., 1998; Carrier et al., 1997; Fukao et al., 1999; Hayabuchi et al., 1998; Stockand and Sansom, 1996; Zhou et al., 2001). Large coronary arteries are more sensitive to Nitric Oxide-cyclic guanosine monophosphate (NO-cGMP) signaling than smaller coronary arteries, due to higher PKG and MYPT1 expressions in larger coronary vessels (Ying et al., 2012). As described above, cGMP-mediated PKG1α stimulation prevents oxidant-induced Cys42 disulfide formation within PKG1α rendering large arteries less sensitive to H_2_O_2_. Furthermore, larger vessels have higher peroxiredoxin (Prx) levels compared with smaller vessels, which could decrease the levels of H_2_O_2_ in contrast to smaller vessels. Therefore, it is likely that smaller vessels are more sensitive to H_2_O_2_-induced Cys disulfide bridge formation in PKG1α and the subsequent H_2_O_2_-induced vasorelaxation (Burgoyne et al., 2012; Prysyazhna et al., 2012). Santiago and colleagues confirmed that the larger proximal coronary arteries resulted in contractions in response to H_2_O_2_, whereas the smaller distal coronary arteries resulting in concentration-dependent relaxations (Santiago et al., 2013).

An important factor that modulates the outcome of vasoactive agents on vascular reactivity is the level of depolarization or V_m_ before relaxation. Under quiescent and noncontracted conditions, exogenous application of H_2_O_2_ constricts mouse abdominal aorta more than the thoracic aorta or carotid arteries but does not constrict the mesenteric artery (Ardanaz and Pagano, 2006). Raising V_m_ with 30 mM K^+^, enhanced contractile responses to H_2_O_2_ and unmasks a contractile response to H_2_O_2_ in the mesenteric artery (Santiago et al., 2013). This suggests that depolarization blocks an underlying hyperpolarization in smaller arteries. In smaller resistance arteries, the role of EDH is more important (Hilgers et al., 2006; Shimokawa et al., 1996) and the density of endothelial K_Ca_3.1 and K_Ca_2.3 channels and myo-endothelial gap junctions initiating and propagating the EDH (*See Section Endothelial K_Ca_ channels*) is more prominent compared with larger arteries (Hilgers et al., 2006; Sandow et al., 2009). H_2_O_2_ has been considered a contributor to EDH-mediated relaxations of coronary and small mesenteric arteries from mice, dogs, and humans (Matoba and Shimokawa, 2003; Matoba et al., 2003; Matoba et al., 2002; Matoba et al., 2000; Miura et al., 2003; Morikawa et al., 2003; Pomposiello et al., 1999; Rubanyi and Vanhoutte, 1986; Shimokawa and Morikawa, 2005; Yada et al., 2003). In most of these studies, oxidative stress is not enhanced and H_2_O_2_ is produced by eNOS and functions as a compensatory endothelium-derived relaxing factor (Fujiki et al., 2005; Kang et al., 2007; Matoba et al., 2000). These observations provide a mechanistic basis for the concept that large conduit artery vasodilation is mediated *via* NO, whereas EDH predominates in resistance vessels (Burgoyne et al., 2012). Intriguingly, in heterozygous GPx1^+/−^ mice, metacholine-mediated dilatations are impaired in microvascular mesenteric arteries (Forgione et al., 2002). In homozygous GPx1^−/−^ mice, responses to ACh were impaired in isolated carotid arteries and Gpx1 protects against Ang II-induced endothelial dysfunction, suggesting that elevated levels of H_2_O_2_ cause endothelial dysfunction (Chrissobolis et al., 2008). Apparently, a delicate threshold and a fine balance of intracellular H_2_O_2_ exist; normal H_2_O_2_ levels promote endothelium-dependent relaxations, whereas higher levels inhibit endothelium-dependent relaxations. Although the threshold of H_2_O_2_ remains unclear and unknown, Prxs play a major role in regulating the baseline levels of H_2_O_2_ in these vessels. Others dismiss a role for H_2_O_2_ in EDH, which may result from intra-artery differences or perhaps the source of catalase tested to scavenge H_2_O_2_ (Beny and von der Weid, 1991; Chaytor et al., 2003; Ellis et al., 2003; Hamilton et al., 2001). Intriguingly, we observed blunted H_2_O_2_-mediated relaxations in small mesenteric arteries from mice overexpressing human Trx (Trx-Tg) (Hilgers and Das, 2015).

### Voltage-dependent K^+^ channels

#### Nomenclature and function

In resistance arteries, VSMC V_m_ is an important regulator of vasomotor tone. Voltage-dependent K^+^ channels (Neckář et al., 2019) are sensitive to changes in V_m_ since depolarization increases and hyperpolarization decreases K_V_ channel activity. V_m_ is determined by the membrane permeability to K^+^, Ca^2+^, Na^+^, and Cl^−^, with K^+^ playing the most important role in regulating VSMC V_m_. K_V_ channels are also known as delayed rectifier or transient outward currents and are expressed throughout the vascular tree (Bonnet et al., 1991; Gelband and Hume, 1992; Okabe et al., 1987; Smirnov and Aaronson, 1992; Volk and Shibata, 1993). Following the discovery of Kv 1.3K^+^ channel in human T cells, several high-affinity inhibitors were developed to block Kv1.3 to inhibit autoimmune diseases (Varga et al., 2021).

#### Structure

The K_V_ channel has a characteristic pore-forming unit composed of six transmembrane segments. The K_V_ channel is assembled by four of these units. The pore region is formed by S5 and S6 and the voltage-sensor domain by S1 to S4. The S4 region of the K_V_ channel containing positively charged residues has been implicated as the voltage sensor (Sigworth, 1994). Functional K_V_ channels assemble as tetramers of pore-forming α subunits (Pongs et al., 1988).

#### Activity and redox modulation

Closure of the pore-forming α subunit depends on occlusion of a “ball like” peptide structure located in the amino terminus of the α subunit into the pore-forming cavity of the α subunit (Hoshi et al., 1990). A soluble peptide containing this ball sequence inhibited Shaker K^+^ channel in a concentration-dependent manner (Zagotta et al., 1990). This inactivation mechanism is redox-dependent such that Cys thiol oxidation causes a disulfide bridge formation between a critical Cys residue (Cys13) in the α subunit and the Cys residue of the ball peptide (Ruppersberg et al., 1991). Loss of this fast-acting inactivation is reversed by the reducing agents GSH and DTT (Ruppersberg et al., 1991). Diamide activates neuronal K_V_4 channels expressed in *Xenopus laevis* oocytes *via* Cys13 S-glutathionylation (Jerng and Pfaffinger, 2014). Apparently, the addition of glutathione to Cys13 results in a marked slowing of N-type inactivation and a concomitant increase in peak current, presumably *via* interfering with this ball-like inhibition. Also, in the mesenteric artery, exogenous application of H_2_O_2_ results in K_V_ channel activation *via* Cys S-glutathionylation, although it was not discerned whether the site of S-glutathionylation occurred at Cys13 (Park et al., 2015). In addition, myocytes isolated from infarcted heart showed increased mRNA and protein expression of Kv4.2 and Kv4.3 channel alpha subunits. Pyruvate-induced increase in Kv4.x expression was blocked by auranofin, and inhibitor of Trx reductase. These data suggested that Kv4.x channel expression is redox regulated by the Trx system (Li et al., 2008b). In A-type K_V_ channel subtypes expressed in neurons, a regulatory β subunit possesses a similar “ball-like” structure in the amino terminus, and its inactivation is dependent on a disulfide bridge formation between Cys residues (Heinemann et al., 1995; Heinemann et al., 1994; Wang et al., 1996). Indeed, SCAM analysis revealed important Cys residues in the S6 region involved in the pore gating mechanism, confirming an important modulatory role for Cys residues in K_V_ channel gating (Liu et al., 1997). This mechanism of inactivation *via* an oxidoreductase activity of K_V_ channels may be an important biological oxidative stress sensor allowing K_V_ channels to switch rapidly between a closed and open state (Bahring et al., 2001a; Bahring et al., 2001b). Cys thiol oxidation by H_2_O_2_ and DTNP has been shown to suppress the K_V_ current in mouse colonic SMCs (Prasad and Goyal, 2004), but to stabilize the open state of K_V_1.4 in *Xenopus laevis* oocytes (Stephens et al., 1996). The redox environment in the cell is more reduced compared with the more oxidized cell-free patch-clamp recording configuration (Schlief et al., 1996; Strupp et al., 1992). This may be a plausible explanation for many differential effects observed studying thiol oxidative agents and K^+^ channel activity. In the coronary circulation, H_2_O_2_ activates K_V_ channels resulting in relaxations, whereas DTT can reverse this H_2_O_2_-mediated coronary vasodilation (Myers et al., 1996). It is believed that Cys thiol modification is a redox sensor in the control of coronary blood flow (Myers et al., 1996). In K_V_2.1 channels, all 15 Cys residues are located on the intracellular side, hence Cys modulation takes place at the cytosolic side, which means that H_2_O_2_ needs to cross the cell membrane when applied exogenously. This may be an explanation why such high concentrations of H_2_O_2_ are needed to promote Kv channel activation since H_2_O_2_ is rapidly degraded by cellular Prxs (Rhee et al., 2001; Wood et al., 2003). K_V_ channels are oxygen-sensitive and play a major role in the pulmonary circulation where oxygen levels are high and cellular redox is in a more oxidized state. Kv 1.3K^+^ channel is a primary target of autoimmune disease (Panyi et al., 2004). Upon antigen presentation, activation of T cells requires Ca+ signal, which requires Ca+ entry to the cytosol through calcium release-activated Ca^2+^ channel (CRAC). Once CRACs are open, the level of Ca^2+^ signal is determined by an electrochemical driving force for Ca^2+^ entry (Feske et al., 2015). The driving force for Ca^2+^ is determined by an interplay between the Ca^2+^ influx *via* Kv1.3 and the intermediate conductance calcium-activated KCa3.1K^+^ channel (Cahalan and Chandy, 2009; Feske et al., 2015).

### Trx system in the pulmonary circulation

#### Redox sensing in the pulmonary artery during normoxia

The pulmonary circulation is a low-pressure and highly compliant vasculature, quite different from the systemic circulation. The lungs are very sensitive to changes in O_2_ tension. In the developing fetus, the pulmonary arteries are exposed to hypoxia and constrict, while the ductus arteriosus (DA) is dilated and acts as a right-to-left shunt pathway to divert blood directly into the systemic circulation (Heymann and Rudolph, 1975). Immediately after birth, O_2_ induces Trx, TrxR, and Prx expression in the lungs of newborn primates, implying an important role for the Trx system in lung maturation and possibly pulmonary artery function (Das et al., 2001; Das et al., 1999). In response to the dramatic change in O_2_ tension, opposite effects on the pulmonary artery and DA occur, namely pulmonary artery vasorelaxation and closure of the DA (Michelakis et al., 2002; Olschewski et al., 2004). In contrast, systemic arteries, such as the renal, coronary, and mesenteric arteries, have opposing effects to the DA, namely a vasorelaxation upon K^+^ channel oxidation. What mediates this abrupt change in pulmonary vasoconstriction into vasodilation at birth? The O_2_ sensor and effector molecules mediating pulmonary artery contraction are resided in the mitochondria and PASMCs. During normoxia, the PASMCs convert glucose into ATP *via* the glycolysis in the mitochondria. NADPH donates electrons to O_2_ and Nox (complexes I and III) generate O_2_^•^**^–^** *via* the electron transfer chain (Chandel and Schumacker, 2000). Mitochondrial SOD converts O_2_^•^**^–^** into H_2_O_2_, which diffuses across the mitochondrial cell membrane into the cytosol. Here H_2_O_2_ causes PKG1α dimerization and thiol oxidation of K_V_ and BK_Ca_ channels (Resnik et al., 2006; Zhang et al., 2012). However, it remains unknown whether upon birth, these dimerizations or oxidations occur, although in a uterine hypoxic environment to normoxic environment is an oxidative environment (3% pO_2_ *vs.* 21% pO_2_).

#### Redox sensing in the pulmonary artery during hypoxia

Pulmonary artery hypoxia has the opposing effects as described above. O_2_ deficiency shuts down mitochondrial electron transfer chain and oxidative phosphorylation. Hence, H_2_O_2_ production is decreased. There is still extensive debate whether an increase or decrease in ROS production occurs during hypoxia (Veit et al., 2015; Weir et al., 2005). It is possible that Nox enzymes in PASMC Noxs are still capable of generating ROS. Glycolysis is shifted toward the pentose phosphate pathway, which increases glucose transport *via* the glucose transporter and converts glucose into glucose-6-phospate (G6P). The enzyme G6P dehydrogenase converts G6P at the expense of NADP^+^ and the formation of NADPH. K^+^ channels are redox sensitive in that NAD(P)H inhibits BK_Ca_ and K_V_ channel opening, whereas NAD(P)^+^ activates these channels (Lee et al., 1994; Park et al., 1995). For a more extensive review of the role of ion channels in hypoxia-induced RONS production in the pulmonary artery, the reader is referred to Veit and colleagues (Veit et al., 2015). Here we focus on Cys thiol redox modulation of K^+^ channels in the pulmonary vasculature. The molecular switch of K_V_ channel gating is Cys thiol/disulfide redox modulation causing conformational changes in the gating machinery (Park et al., 1995; Reeve et al., 1995; Yuan et al., 1994). The Cys thiol oxidizer diamide is a potent inhibitor of pulmonary arterial constriction in response to hypoxia and prostaglandin F_2α_ in intact dogs (Schach et al., 2007; Weir et al., 1983).

While the Trx system has been extensively investigated in lung diseases and epithelial cells (Das, 2004; Tipple, 2014), its role in modulating ion channel gating in the pulmonary vasculature is underexplored (Olschewski and Weir, 2015). Since mitochondrial Noxs utilize NADPH, and TrxR uses NADPH to reduce oxidized TrxR into reduced TrxR, the Trx system is expected to play an important role in controlling pulmonary vascular regulation. Interestingly, Burgoyne and colleagues observed that inhibition of TrxR with auranofin increased PKG1α dimerization in human embryonic kidney cells (Burgoyne et al., 2007). Treatment of bovine pulmonary arteries with Trx siRNA increased PKG1α dimerization during hypoxia (Neo et al., 2013). These data suggest that loss of Trx or oxidation of Trx promotes PKG1α disulfide formation. However, more conclusive studies are needed to establish these reasonings.

In the pulmonary artery, voltage-independent transient receptor potential cation (TRPC) channels are widely expressed. Especially the TRPC6 channel is highly expressed in PASMCs (Dietrich et al., 2005). Hypoxia triggers diacylglycerol (DAG) (Tang et al.)-induced TRPC6 channel opening leading to Na^+^ and Ca^2+^ influx, which inhibits K_V_ channels and stimulates pulmonary contraction (Hofmann et al., 1999). Mice deficient in TRPC6 show no acute pulmonary vasoconstriction in response to hypoxia (Weissmann et al., 2006). The TRPC6 channel is inhibited by the NO/cGMP/PKG pathway (Takahashi et al., 2008). Cys thiol oxidation by N-ethylmaleimide or H_2_O_2_ activates the TRPC6 channel and sensitizes it even more to DAG. Cys sulfhydryl reducing agents such as DTT and GSH decrease the activity of TRPC6 (Graham et al., 2010). It is likely that Trx could regulate the activity of TRPC6 channel by modulating redox-active Cys of TRPC6 channel during hypoxic pulmonary vasoconstriction, where it may regulate the Cys disulfide–thiol exchange. Xu and colleagues have shown that Trx acts by breaking a disulfide bridge in the predicted extracellular loop adjacent to the ion-selectivity filter of TRPC5 expressed in human fibroblast-like synoviocytes, thereby opening the channel (Xu et al., 2008). The double-mutant C553A/C558A in TRPC5 was unresponsive to DTT. This modulation of Trx is different from the TRPC6 modulation where disulfide reduction favors channel closing.

### Redox regulation of K^+^ channel and role of Trx in the heart

Cardiac K^+^ channels play an important role in repolarization of the action potential during a heartbeat. In fact, many channel-mediated currents are involved and are reviewed elsewhere (Li and Dong, 2010; Tamargo et al., 2004). All cardiac K^+^ channels are affected by RONS, most of them experience decreased repolarization currents (Matsuura and Shattock, 1991). Abnormal K^+^ channel activity has been linked to electrical remodeling, resulting in patients with heart failure (Armoundas et al., 2001; Rozanski and Xu, 2002a; Rozanski and Xu, 2002b). In ventricular myocytes, it has been shown that the inhibition of K^+^ currents is sufficient to delay repolarization, causing prolongation of the action potential duration, and thus affects myocyte contraction and may lead to cardiac arrhythmias (Pallandi et al., 1987; Tarr et al., 1994). Redox control of these cardiac K^+^ channels by ROS has been extensively reviewed by Aggarwal and Makielski (Aggarwal and Makielski, 2013). Many cardiac ion channels, such as L-type voltage-operated Ca^2+^ channels (L-VOCC) (Campbell et al., 1996; Chiamvimonvat et al., 1995; Lacampagne et al., 1995; Sims and Harvey, 2004), K_IR_ (Gomez et al., 2014), K_ATP_ (Coetzee et al., 1995; Ichinari et al., 1996; Yan et al., 2009), and K_V_ (Hool, 2004; Kolbe et al., 2010; Li et al., 2006b; Rozanski and Xu, 2002b) involved in myocyte contraction, apoptosis, and inflammation, are sensitive to thiol/disulfide protein modification by exogenous oxidoreductases. The abovementioned studies mainly use chemical sulfhydryl reducing or oxidizing agents, such DTT, the oxidizers DTNB and thymidine-5′-diphosphate (DTDP), or 1,3-bis-(2-chloroethyl)−1-nitrosourea (BCNU), to modify Cys thiol/disulfides on K^+^ channels. Atrial fibrillation (AF) is the most common cardiac arrhythmia where ultrarapid delayed rectifier current (I_kur_) is the predominant repolarizing K^+^ current in the atria (Svoboda et al., 2012). This current is encoded by Kv 1.5 channel subunit, which in the human heart is selectively expressed in the atria and is considered an important therapeutic target for treatment of AF (Ford and Milnes, 2008; Svoboda et al., 2012; Wang et al., 1993). This Kv 1.5 channel is an important target for the treatment of AF. Multiple K^+^ channels, including Kv1.5 encoded I_kur_, are regulated by oxidizing agents (Svoboda et al., 2012). Redox-sensitive posttranslational modifications of Kv 1.5 are a major mechanism of regulation of this channel (Poole et al., 2004; Svoboda et al., 2012). Reversible oxidative modification of Cys residue to Cys-sulfenic acid (Cys-SOH) is an important mechanism of protein function in response to modulation of cellular redox state (Poole et al., 2004). Kv 1.5 has 6 intracellular Cys within the NH_2_ and COOH termini (Svoboda et al., 2012). Svoboda et al. have shown a global increase in sulfenic acid-modified proteins in the atria of human patients with chronic AF (Svoboda et al., 2012). They have also shown that Kv 1.5 is a substrate for sulfenic acid modification, which functions as a switch for turning the channel from a recycling mode to a degradation pathway in myocytes. Furthermore, they showed that sulfenic acid modification of COOH terminal C581 alone is sufficient to trigger internalization and degradation of Kv1.5 (Svoboda et al., 2012).

Human Trx can be taken up by cardiomyocytes (Tao et al., 2004) and injection of recombinant human Trx in rats before subjection to myocardial ischemia/reperfusion injury reduced myocardial infarct size, myocyte apoptosis, and inflammation (Wu et al., 2008). We recently reported that vascular Trx overexpression protected against Ischemia-Reperfusion (I/R)-induced myocardial infarction and impairment in coronary artery relaxation *via* deglutathionylation of eNOS (Subramani et al., 2016). Trx interacts with apoptosis signal-regulated kinase-1 (ASK-1), a mitogen-activated protein kinase kinase kinase. Trx-(SH)_2_ binds to ASK-1 and inhibits its activity (Saitoh et al., 1998). Binding of ASK-1 prevents activation of c-Jun NH_2_-terminal kinase (JNK)/p38 MAPK-induced apoptosis pathway. Proapoptotic stimuli such as tumor necrosis factor-alpha and ROS can oxidize Trx-(SH)_2_ into Trx-S_2_ to dissociate Trx from ASK-1 (Liu and Min, 2002). JNK and p38 MAPKs are key regulators of cardiac hypertrophy and apoptosis *via* modulation of K_V_ channels by Trx (Tang et al., 2011). During heart failure, decreased interaction of Trx with ASK-1 induces K_V_ channel downregulation, which is the underlying mechanism of the decreased transient-outward K^+^ current (*I*_to_) (Armoundas et al., 2001; Kaab et al., 1998; Liang et al., 2008). Downregulation of the *I*_to_ current is restored by Trx, which induces the expression of K_V_4.x channels (Li et al., 2008b). Furthermore, these studies suggest that the expression of Kv4.x is redox regulated by Trx although more work is needed to mechanistically establish the role of Trx at a molecular level. In diabetes, which is associated with elevated levels of Cys disulfide bridge formation in cellular proteins (Bidasee et al., 2003), *I*_to_ is reduced due to decreased TrxR activity (Li et al., 2005). K^+^ channels involved in *I*_to_ have one or more Cys sulfhydryl groups that directly control channel function (Rozanski and Xu, 2002b) and hence are susceptible to Trx redox modulation. Recently, H_2_S has been shown to target the Cys320/Cys529 motif in K_V_4.2 channels by breaking the disulfide bond to regulate the I_to_ current in cardiomyocytes (Ma et al., 2015).

K_ATP_ channels are highly expressed in the heart with the K_IR_6.2/SUR2A isoform being the predominant K_ATP_ channel type expressed in cardiac sarcolemma (Akrouh et al., 2009). As described above, ATP inactivates and hypoxia (↓ATP) activates *I*_KATP_ current. Thus, K_ATP_ channels serve as metabolic sensors in the cell that play a major role in protecting the heart from ischemia/reperfusion-induced damage (Garlid et al., 2009). Not surprisingly, the K_ATP_ channel is modulated by Cys thiol modulators, since GSSG, p-chloromercuriphenyl sulfonate (pCMPS), DTNB, thimerosal, and H_2_O_2_ increase *I*_KATP_, which can be reversed by DTT and GSH (Coetzee et al., 1995; Ichinari et al., 1996; Yan et al., 2009).

### Trx and K^+^ channel modulation in the central nervous system

Several neuronal ion channels and receptors are modulated by redox modulation that regulates excitability of neurons (Gamper et al., 2006). BK channels (Tang et al., 2004), heteromeric Iks-like K^+^ channels (Busch et al., 1995), ASIC channels (Andrey et al., 2005), and NMDA receptors (Aizenman et al., 1989) are inhibited by millimolar concentrations of oxidizing agents and GIRK K^+^ channels are activated by reducing agents (Zeidner et al., 2001). Effects of redox on the kinetics of several Kv channels and Human Ether-a-go-go-related Gene (HERG) channels have also been reported (Gamper et al., 2006). A major conceptual advance in this field was made by the demonstration of mechanism of neuroprotective silencing in hypoxia by showing that inwardly rectifying K_ATP_ channels assembled from the pore-forming Kir6.2 and auxiliary SUR subunits are augmented by endogenous H_2_O_2_ (Gamper et al., 2006). Another study has also shown a strong increase in M-channel activity by H_2_O_2_. Consistent with these, H_2_O_2_ was also shown to increase Kv7.2, 7.4, and 7.5 current (Gamper et al., 2006).

High levels of Trx and TrxR are present in neurons and their axons (Rozell et al., 1985). Trx mRNA has been observed in regions involved in neuroendocrine and/or cardiovascular control such as the paraventricular hypothalamic nucleus and the nucleus of the solitary tract (Lippoldt et al., 1995). Interestingly, Trx levels are higher in neurons compared with GSH levels (Patenaude et al., 2005). Furthermore, the Trx system has many neuroprotective effects in the brain where it plays a crucial role in combating increases in oxidative stress (Silva-Adaya et al., 2014). Another member of the Trx family is the macrophage migration inhibitory factor (MIF) (Bloom and Bennett, 1966). MIF is constitutively expressed in neurons within the brain (Bacher et al., 1998). The oxidoreductase activity of MIF is exerted by Cys57 and Cys60 (Kleemann et al., 1998), and one potential consequence of an increase in oxidoreductase activity is scavenging of ROS and blockade of oxidant-mediated intracellular actions (Nguyen et al., 2003; Sun et al., 2004). Acting *via* Ang II type 1 (AT_1_) receptors in hypothalamic paraventricular nucleus neurons, Ang II increases sympathetic activity and blood pressure (Ferguson et al., 2001; McKinley et al., 2003; Oparil et al., 1994). Increased rates of sympathetic nerve firing and reduced neuronal norepinephrine reuptake both contribute to sympathetic activation in hypertension (Schlaich et al., 2004). MIF intracellularly decreases basal neuronal firing and counteracts the AT_1_ receptor-mediated chronotropic effects of Ang II in hypothalamic neurons (Li et al., 2008a; Li et al., 2006a; Sun et al., 2004) *via* its oxidoreductase activity (Matsuura et al., 2006). This is an important observation with regard to the mechanism of neurogenic hypertension. Changes in neuronal outward K^+^ current contribute to changes in neuronal firing (Sun et al., 2005). Voltage-gated potassium (Neckář et al., 2019) channels, mediating this neuronal outward K^+^ current or delayed-rectifier K_V_ currents (*I*_KV_), are widely expressed in the central and peripheral nervous system (Misonou et al., 2005). K_V_ channels help generate action potentials (depolarization causes K_V_ channel activation) as well as maintaining the resting V_m_ of neurons (hyperpolarization or repolarization closes the K_V_ channel). Increased *I*_KV_ currents inhibit the neuronal firing. Ang II has been shown to reduce the *I*_KV_ current and to decrease the activity of a 4-AP-sensitive K_V_ channel in neurons (Wang et al., 1997a). Intracellular application of MIF (80 nM) or Trx (0.8–80 nM) into rat hypothalamic/brain stem neurons in culture increased neuronal *I*_KV_, as measured by voltage-clamp recordings (Matsuura et al., 2007; Matsuura et al., 2006). It can be concluded from these studies that sulfhydryl modulation regulates basal and stimulated neuronal activity by increasing neuronal *I*_KV_ within the hypothalamus and brain stem, since the mutant C32S/C35S Trx was ineffective (Matsuura et al., 2007).

Thiol oxidation of K_V_2.1 channels by DTDP induces an intracellular release of Zn^2+^ from metal-binding proteins, which is required for K_V_2.1 channel activation. This triggers a long-lasting enhancement of outward K^+^ current promoting neuronal apoptosis, contributing to widespread neuronal loss in neurodegenerative disorders such as Parkinson’s disease, Alzheimer’s disease, and stroke (Aizenman et al., 2000; McBean et al., 2015; McLaughlin et al., 2001; Shah and Aizenman, 2014; Yu et al., 1997). Neuronal apoptosis is dependent on ASK-1 (Saitoh et al., 1998). Trx is able to inhibit ASK-1 and halt the apoptotic current surge in neurons (Aras and Aizenman, 2005). However, whether Trx can reduce disulfides in Kv2.1 channels is unclear. The importance of Cys residues in K_V_ channels has been well established (see the Voltage-Dependent K^+^ Channels section). Many K_V_ channels contain two conserved Cys residues in putative transmembrane segments S2 and S6. It has been proposed that these Cys form an intrasubunit disulfide bond (Guy and Conti, 1990). Using a mutated K_V_2.1 channel or creating a Cys residue at 379 resulted in H_2_O_2_-induced disulfide bridge formation with the adjacent Cys394 locking the channel in a permanent nonconducting state (Zhang et al., 1996). The conserved Cys393 in S6 is positioned in a region of tight protein packing adjacent to the ion-conduction pathway (Zuhlke et al., 1994). This Cys residue participates in pore gating by stabilizing the open state (Liu and Joho, 1998). The sulfhydryl oxidizing agent thimerosal interacts with Cys214 in the K_V_7.1 or KCNQ1 channel resulting in a reduced current (Kerst et al., 2002). Neuronal Kv7 channels are important regulators of cell excitability, which are sensitive to ROS. The S2S3 linker of the voltage sensor was reported to be sensitive to redox regulation (Nunez et al., 2023). Cys residues in the S2S3 loop relieve Kv7 channel from constitutive inhibition by interaction between EF3 hand of CaM, which is crucial for redox regulation of Kv channel (Nunez et al., 2023). Another K_V_ channel type, K_V_10.1, is widely expressed in the central nervous system. Interestingly, oxidative modification of K_V_10.1 channel is governed at the Cys pairs (Cys145/Cys214) situated at structural elements in the K_V_10.1 channel involved in channel gating (Sahoo et al., 2012). These observations suggest a potential modulatory role for Trx in reducing disulfide bridges in K_V_ channels in the central nervous system.

### Redox regulation of Kvβ channels

Kv pore-forming complex is composed of four distinct subunits (Kvα) that form the voltage-sensing, gating, and selectivity domains (Long et al., 2005; Matthies et al., 2018, Moran et al., 2015). Kvα pore proteins are linked to several types of cytoplasmic protein complexes, which regulate subcellular localization and gating kinetics (Long et al., 2005; Pongs and Schwarz, 2010). Kvβ proteins are associated with Kv1 and Kv4 families, which are aldo-keto reductases (AKRs) that catalyze the NADPH-dependent reduction carbonyl substrates to primary and secondary alcohols (Dwenger et al., 2022). Furthermore, Kvβ AKR activity has been shown to regulate channel trafficking and gating (Dwenger et al., 2022). In addition, it has been shown that Kvβ proteins catalyze reduction of carbonyl substrates *via* hydride transfer from NADPH (Mindnich and Penning, 2009). Thus, Kvβ proteins represent a molecular link between the cellular redox state and membrane potential regulation (Dwenger et al., 2022). Previous studies have suggested that Kvβ2 specifically uses NADPH as reducing equivalents for reduction (Tipparaju et al., 2008; Weng et al., 2006). However, the physiological carbonyl substrates for Kvβ and their impact on channel function have not been identified. Several *in vitro* studies show that purified Kv2 reduces a range of endogenous and exogenous carbonyls (Dwenger et al., 2022). Along with C-nitro compounds, Kvβ 2 readily reduces phenanthrenequinone, glycolytic by-product methylglyoxal, and oxidized phospholipids (1-palmitoyl-2-oxovaleroyl phosphatidylcholine and 1-palmitoyl-2-arachidonoyl-sn-glycero-3-phosphocholine (PAPC) (Dwenger et al., 2022; Xie et al., 2011). The finding that Kvβ reduces the products of PAPC is consistent with the notion that Kvβ AKR activity may be responsible for membrane oxidative stress under physiological or pathological conditions (Dwenger et al., 2022).

### K^+^ channels as therapeutic target

The discovery of the Kv1.3 voltage-gated K^+^ channel in human T cells in 1984 (Chandy et al., 1984), promoted the role of ion channels as important signal transducers in the immune system (Varga et al., 2021). Targeting the Kv1.3 potassium channel has been shown to decrease obesity and the severity of animal models of autoimmune disease. The voltage-gated Kv1.3 potassium channel has recently been targeted for obesity control, as mice lacking Kv1.3 or treated with a Kv1.3 blocker are protected from high-fat diet-induced obesity (Upadhyay et al., 2013; Xu et al., 2003). Kv1.3 is also a target for autoimmune diseases, as C-C chemokine 7 (CCR7)^-^ effector memory T (Wedmann et al.) lymphocytes express high levels of Kv1.3 upon activation, and blocking of Kv1.3 inhibits their function (Chandy and Norton, 2017).

Cardiac K^+^ channels play an important role in maintaining the normal electrical activity of the heart by setting the cell resting membrane potential and by determining the shape and duration of the action potential (Walsh, 2015). Drugs that block the rapid (Steudel et al., 1999) and slow (Sahlin et al.) components of the delayed rectifier K^+^ current have been widely used as class III antiarrhythmic agents (Walsh, 2015). In addition, drugs that selectively target the ultrarapid delayed rectifier current (I_Kur_) and the acetylcholine-gated inward rectifier current (I_KAch_) have shown efficacy in the treatment of patients with AF. To meet the future demand for new antiarrhythmic agents, novel approaches for cardiac K^+^ channel drug discovery will need to be developed. Furthermore, K^+^ channel screening assays utilizing primary and stem cell-derived cardiomyocytes will be essential for evaluating the cardiotoxicity of potential drug candidates. Small-conductance Ca^2+^-activated K^+^ (SK)-channel inhibitors have antiarrhythmic effects in animal models of AF, presenting a potential novel antiarrhythmic option. I_SK_ is upregulated in patients with chronic AF due to enhanced channel function, mediated by phosphatase-2A-dependent CaM-Thr80 dephosphorylation and tachycardia-dependent enhanced trafficking and targeting of SK-channel subunits to the sarcolemma (Walsh, 2015). The observed AF-associated increases in I_SK_, which promote reentry-stabilizing action potential duration shortening, suggest an important role for SK-channels in AF auto-promotion and provide a rationale for pursuing the antiarrhythmic effects of SK-channel inhibition in humans (Walsh, 2015).

Dysfunctions of K_Ca_2.3 and K_Ca_3.1 that are associated with membrane hyperpolarization and EDH-mediated dilation are implicated in cardiovascular pathologies, including diabetes, atherosclerosis, inflammation, cancer, and fibrosis (Grgic et al., 2009; Kohler et al., 2016). In addition, K_Ca_3.1 plays an important role in endothelial barrier dysfunction, edema formation in cardiac and pulmonary disease, and in stroke. Genome-wide association studies revealed an association of K_Ca_2.3 channels with AF in humans. Accordingly, both channels are considered potential drug targets for cardiovascular diseases.

K_ATP_ channels are targets for antidiabetic sulfonylurea blockers, and for channel opening drugs that are used as antihypertensives (Rodrigo and Standen, 2005). In vascular smooth muscle, K_ATP_ channels are extensively regulated by signaling pathways and cause vasodilation, contributing both to resting blood flow and vasodilator-induced increases in flow (Rodrigo and Standen, 2005). Similarly, K_ATP_ channel activation relaxes smooth muscle of the bladder, gastrointestinal tract, and airways (Rodrigo and Standen, 2005). In cardiac muscle, sarcolemmal K_ATP_ channels open to protect cells under stress conditions such as ischemia or exercise, and appear central to the protection induced by ischemic preconditioning (IPC) (Rodrigo and Standen, 2005). Mitochondrial K_ATP_ channels are also strongly implicated in IPC (Rodrigo and Standen, 2005). Skeletal muscle K_ATP_ channels play roles in fatigue and recovery, K^+^ efflux, and glucose uptake. Current therapeutic considerations include the use of K_ATP_ openers to protect cardiac muscle (Rodrigo and Standen, 2005), attempts to develop openers selective for airway or bladder, and the question of whether block of extra-pancreatic K_ATP_ channels may cause adverse cardiovascular side effects of sulfonylureas (Rodrigo and Standen, 2005). Congenital hyperinsulinism (HI), is a beta cell disorder caused by inactivating mutations of beta cell K_ATP_ channels, results in dysregulated insulin secretion and persistent hypoglycemia (Juliana et al., 2023). Children with K_ATP_-HI are unresponsive to diazoxide, the only FDA-approved drug for HI, and utility of octreotide (McMahon et al., 2017), the second-line therapy, is limited because of poor efficacy, desensitization, and somatostatin receptor type 2 (SST2)-mediated side effects. A recent study has shown that CRN02481, a highly selective nonpeptide SST5 agonist, significantly decreased basal and amino acid-stimulated insulin secretion in both Sur1^−/−^ (a model for K_ATP_-HI) and wild-type mouse islets. Oral administration of CRN02481 significantly increased fasting glucose and prevented fasting hypoglycemia compared with vehicle in an HI mouse model (Sur1^−/−^ mice) (Juliana et al., 2023). During a glucose tolerance test, CRN02481 significantly increased glucose excursion in both WT and Sur1^−/−^ mice compared with the control. CRN02481 also reduced glucose- and tolbutamide-stimulated insulin secretion from healthy, control human islets such as the effects observed with SS14 and peptide somatostatin analogs (Juliana et al., 2023). Moreover, CRN02481 significantly decreased glucose- and amino acid-stimulated insulin secretion in islets from two infants with K_ATP_-HI and one with Beckwith–Wiedemann syndrome-HI (Juliana et al., 2023).

#### Selenium deficiency and disease

Selenium is a trace element and present in some natural foods and available as a dietary supplement. Selenium is in the active catalytic center of antioxidant enzymes such as TrxR and in glutathione peroxidase. These catalytic centers are responsible to regenerate thiols from Trx disulfide and neutralize various peroxides and function as antioxidants. However, clinical trials with selenium supplementation were not conclusive and provided contradictory results (Bleys et al., 2009; Hercberg et al., 2004). Serum concentrations decline with age (Akbaraly et al., 2007). Therefore, selenium deficiencies could promote age-related accumulation of oxidized products in the body and may cause accelerated aging and malfunction of antioxidant systems. Deficient selenium levels might be associated with age-related decline in brain function due to decrease in selenium’s antioxidant activities (Akbaraly et al., 2007). However, results of human observational studies are mixed (Loef et al., 2011). Taken together, more studies regarding the beneficial effects of supplemental selenium are needed. However, selenium is required to maintain some of the antioxidant enzyme function, but high levels of selenium may not be necessary as selenium in a minute quantity would perform its antioxidant function; therefore, selenium containing food should be adequate for enzymatic activity of antioxidants.

### Future research directions

Almost all K^+^ channels contain reactive Cys in critical regions that regulate pore opening, closing, and regulating polarization and hyperpolarization, which not only impacts channel structure and function, but also allows for redox regulation of these channels in response to oxidative stress or drug-related activation/inhibition. Posttranslational Cys sulfhydryl modulations are physiologically reversible processes (such as phosphorylation/dephosphorylation) that are in perfect equilibrium in a healthy cellular environment. At advanced stages of oxidative stress where Trx, Grx, and Prx activities are oxidized to a certain extent, irreversible Cys thiol modifications could alter protein activities. Aging is an example of a progressive and chronic oxidative process where accumulation of oxidative proteins promotes cardiovascular dysfunction such as hypertension, cardiac dysfunction, atherosclerosis, and many others. We recently showed that overexpression of human Trx1 in mice protected against age-related hypertension *via* reversal of uncoupled eNOS that generates O_2_^•^**^–^** to functional eNOS that produces NO (Das et al., 2018; Hilgers and Das, 2015; Hilgers et al., 2017). We further showed that age-related hypertension is reversed by the treatment of aged mice with recombinant human Trx1, demonstrating a potential therapeutic efficacy of Trx in hypertension and potentially in many other cardiovascular diseases (Das et al., 2018; Hilgers et al., 2017). Given the abundance of Cys residues in proteins/enzymes, the potential for Cys oxidoreductase modulation by the Trx system is enormous and the Trx system is anticipated to play many more beneficial roles in maintaining normal physiology.

## Abbreviations Used

AA: arachidonic acid
AF: atrial fibrillation
AKRs: aldo-keto reductases
ASK1: apoptosis signal-regulated kinase-1
AT1: angiotensin II type I receptor
BCNU: 1,3-bis-(2-chloroethyl)-1-nitrosourea
BK_Ca_: large-conductance Ca^2+^-activated K^+^ channels
CAM: calmodulin
CaMBD: calmodulin binding protein
CRAC: calcium-release activated Ca^2+^ channel
cGMP: guanosine 3′-5′-cyclic monophosphate
Cys: cysteine
DAG: diacylglycerol
DTDP: thymidine-5′-diphosphate
DTNB: 5,5′-dithio-bis (2-nitrobenzoic acid)
DTT: dithiothreitol
DA: ductus arteriosus
*E. Coli* Trx: *Escherichia coli* Trx
EC: endothelial cell
EDH: endothelium-dependent hyperpolarization
eNOS: endothelial nitric oxide synthase
G6P: glucose-6-phospate
Gly: glycine
Gpx-1: glutathione peroxidase-1
GSH: reduced glutathione
GSSG: oxidized glutathione
GYG: Glycine-Tyrosine-Glycine
H_2_O_2_: hydrogen peroxide
H_2_S: hydrogen sulfide
HCy: homocysteine
HHCy: hyperhomocysteinemia
HI: hyperinsulinism
IK_Ca_: intermediate conductance K_Ca_
IPC: ischemic preconditioning
K^+^ channel: potassium channel
K_ATP_: ATP-sensitive and K^+^ channels
K_Ca_: calcium-activated potassium channel
KCNMB1-4: potassium calcium-activated channel subfamily M regulatory beta subunit 1-4
KcsA: bacterial K^+^ channel
KIR: inward rectifier channel
KV: voltage-dependent K^+^ channel
L-VOCC: L-type voltage-operated calcium channels
LINGO2: leucine-rich repeat and Ig domain containing 2
MIF: migration inhibitory factor
MTS: methanethiosulfonate
MTSEA: 2-aminoethyl methanethiosulfonate hydrobromide
MTSES: sodium (2-sulfonatoethyl) methanethiosulfonate
MTSET: [2-(trimethylammonium) ethyl] methanethiosulfonate bromide
MYPT1: myosin phosphatase target subunit 1
Na^+^/K^+^: ATPase-sodium/potassium adenosine triphosphatase
NADP: nicotinamide adenine dinucleotide phosphate (oxidized form)
NADPH: nicotinamide adenine dinucleotide phosphate (reduced form)
O_2_.^−^: superoxide anion
ONOO^−^: peroxynitrite
PAPC: 1-palmitoyl-2-arachidonoyl-sn-glycero-3-phosphocholine
PASMC: pulmonary artery smooth muscle cell
PCMBS: parachloromercuribenze sulfonate
pCMPS: p-chloromercuriphenyl sulfonate
PKG1α: protein kinase G alpha
Pro: proline
Prx: peroxiredoxin
RCK1: regulator of K^+^ conductance 1
RONS: reactive oxygen and nitrogen species
ROS: reactive oxygen species
SCAM: substituted cysteine accessibility mutagenesis
SK_Ca_: small conductance K_Ca_
SpTrx: spermatozoa Trx
SUR: sulfonylurea receptor
Thimerosal: [(O-carboxyphenyl)thio]ethyl mercury sodium salt
TNFα: tumor necrosis factor-alpha
TRPC: transient receptor potential cation
Trx: thioredoxin (cytosolic)
Trx-S2: oxidized form of Trx
Trx-SH: reduced form of Trx
TrxR1: thioredoxin reductase-1
VSMC: vascular smooth muscle cell
